# Conazoles

**DOI:** 10.3390/molecules15064129

**Published:** 2010-06-09

**Authors:** Jan Heeres, Lieven Meerpoel, Paul Lewi

**Affiliations:** 1 Leemskuilen 18, B-2350 Vosselaar, Belgium; E-Mail: jan@heeres.be (J.H.); 2 Johnson & Johnson Pharmaceutical Research & Development, a division of Janssen Pharmaceutica N.V.,Turnhoutseweg 30, B-2340 Beerse, Belgium; 3 Pater Van Mierlostraat 18, B-2300 Turnhout, Belgium; E-Mail: paul.lewi@hotmail.com (P.L.)

**Keywords:** miconazole, ketoconazole, itraconazole, posaconazole, fluconazole, voriconazole, antifungal

## Abstract

This review provides a historical overview of the analog based drug discovery of miconazole and its congeners, and is focused on marketed azole antifungals bearing the generic suffix “conazole”. The antifungal activity of miconazole, one of the first broad-spectrum antimycotic agents has been mainly restricted to topical applications. The attractive *in vitro* antifungal spectrum was a starting point to design more potent and especially orally active antifungal agents such as ketoconazole, itraconazole, posaconazole, fluconazole and voriconazole. The chemistry, *in vitro* and *in vivo* antifungal activity, pharmacology, and clinical applications of these marketed conazoles has been described.

## Abbreviations

C.a.: *Candida albicans*
CMC: Chronic mucocutaneus candidiasis
C.tr.: *Candida tropicalis*
A.f.: *Aspergillus fumigatus*
AIDS: Acquired Immune-Deficiency Syndrome
Cr.n.: *Cryptococcus neoformans*
CYP: Cytochrome P-450
EMEM: Eagle’s Minimum Essential Medium
HIV: human immune-deficiency virus
MIC_90_: Minimum inhibitory concentration that inhibited 90% of the test isolates
M.c.: *Microsporum canis*
MIC: Minimum inhibitory concentration
Muc.: *Mucor* species
Ph.v.: *Phialophora verrucosum*
Sap.: *Saprolegnia*
Sp.s.: *Sporothrix schenckii*
Spp.: species
T.m.: *Trichophyton mentagrophytes*
T.r.: *Trichophyton rubrum*

## 1. Introduction

**Figure 1:**
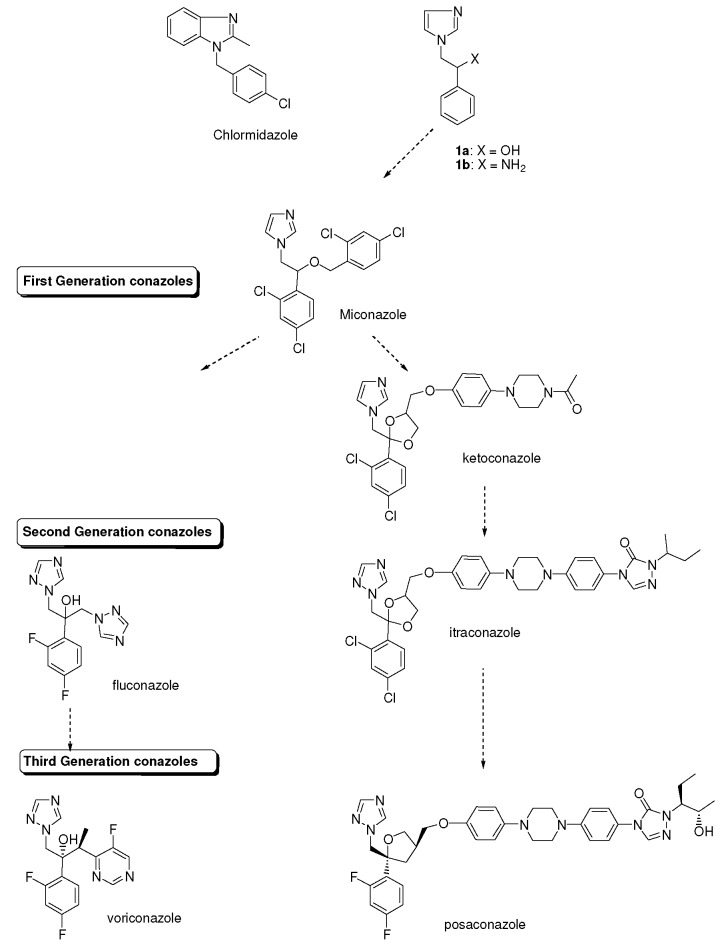
Conazoles.

About fifty years ago a research program focussed on imidazole chemistry was initiated at Janssen. There were a number of reasons to start with imidazole chemistry. First of all because the imidazole moiety is present in the neurotransmitter histamine, which in turn is a metabolite of the amino acid histidine. Moreover chlormidazole (Figure 1), a benzimidazole derivative, displayed antifungal activity.

In this period imidazole chemistry was rather unexplored and therefore it was necessary to find favourable reaction conditions to carry out *N*-alkylations in the 1-position of the imidazole ring without causing quaternization.

Our first objective was to find an acceptable synthesis route for the preparation of *N*-phenacyl imidazoles, because this type of molecules gave access to a library of diversified compounds. Protected ketone derivatives generated opportunities to do reactions on the imidazole ring.

A broad-screening program at Janssen revealed the antifungal activity of some imidazole derivatives. This discovery gave rise to explore opportunities we had generated with *N*-phenacyl imidazole as starting material.

In that period only griseofulvin, tolnaftate and nystatin were available as serious treatments for superficial fungal infections caused by dermatophytes and yeasts, respectively. On the other hand amphotericin B was available for intravenous treatment of systemic deep mycoses. Both nystatin and amphotericin B are polyene antibiotics. In those days market potential for antifungals was small, with estimates of a few million dollars. On the other hand some expert mycologists gave indications that at that time fungal infections were being under-treated or not treated at all. These people pointed out that there was a medical need to find potent antimycotic agents, which not only were able to treat superficial infections but also the emerging deep mycoses which were life-threatening. Moreover they expected the market would grow and they were right, as the market for antifungals now has a potential of 3-5 billion dollars.

The incidence of life-threatening fungal infections in hospitals in the United States has steadily increased. According to the National Nosocomal Infection Surveillance System a total of 30,477 episodes of fungal infections were recorded in the period from 1980 to 1990. Data collected from 49 hospitals over a 7-year period (1995-2002) revealed that *Candida* species (9%) had become the fourth most common cause of infection, following nosocomial bacteraemia caused by coagulase-negative staphylococci (31.3%), *Staphylococcus aureus* (20.2%), and *Enterococcus* species. Notwithstanding that *Candida albicans* is responsible for the majority of infections, there is a trend indicating a shift toward infections by non-albicans *Candida* species, *Aspergillus* species, and previously uncommon opportunistic fungi like filamentous fungi such as *Fusarium* species, a large variety of dematiaceous moulds, zygomycetes such as *Mucor* species, and dimorphic fungi such as *Coccidioides immitis* [171,172]. In severe immunocompromised patients, primary transplant recipients, patients with hematologic malignancies receiving chemotherapy, and HIV-infected patients (AIDS) these infections are increasingly problematic [1].

This review focuses on the discovery, synthesis, SAR, pharmacology, pharmacokinetics and clinical results of the antifungal conazoles. The content is divided into a part dedicated to compounds, which are mainly used for topical treatment of superficial fungal infections, and another part dedicated to compounds which are suitable for oral treatment of superficial and deep life-threatening mycoses.

Miconazole, mainly used for topical treatment of fungal infections, is the first member of the conazole pedigree and so far posaconazole is the latest member of the family on the market and can be considered as a valuable addition to the therapeutic armamentarium against systemic life-threatening fungal infections.

Notwithstanding the convincing topical antifungal activity of clotrimazole, flutrimazole and bifonazole we have resticted our attention to the generic class of the conazoles, which have made the evolution from topical to oral applicable antifungal agents and are responsible for saving lives of critically ill patients infected with life-threatening fungal agents.

## 2. Syntheses and Structure-Activity-Relationship

**Scheme 1:**
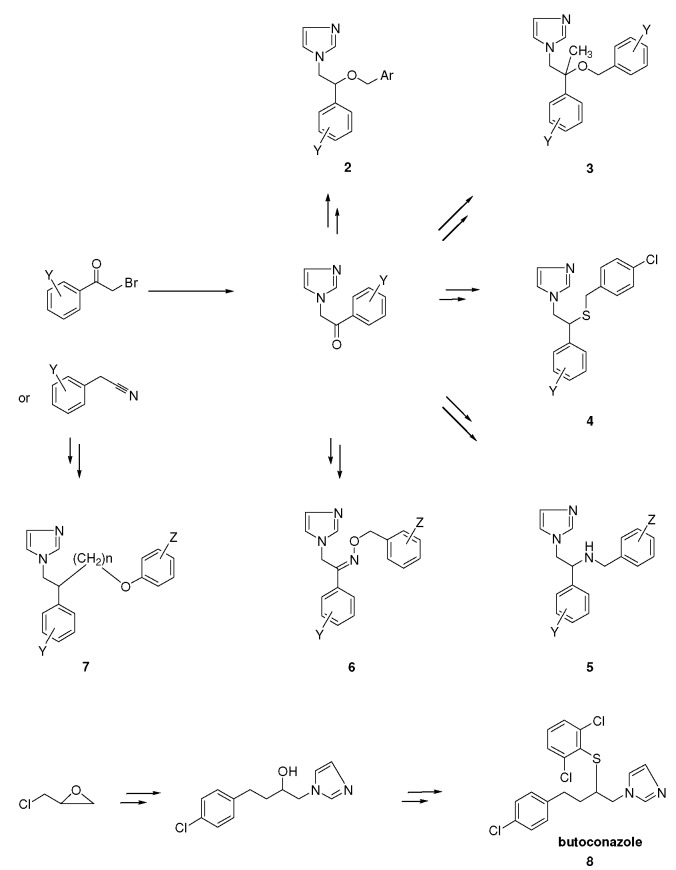
Synthesis of miconazole and analogues [2].

**Figure 2:**
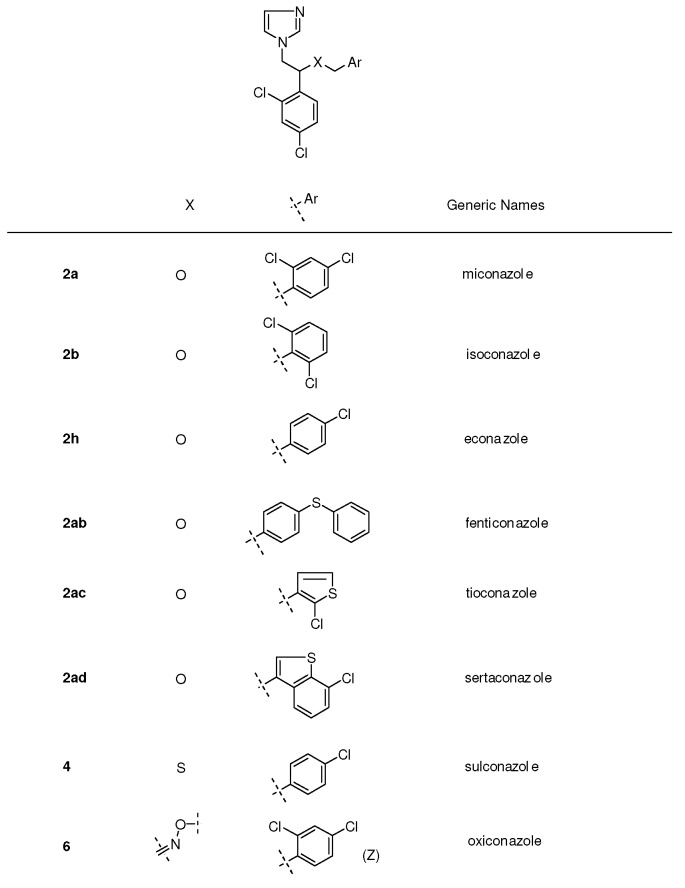
Miconazole and marketed analogues.

It turned out that after screening a series of 1-phenethylimidazoles, in particular O- and N-substituted derivatives of α-phenylimidazole-1-ethanol, (**1a**, X=OH) and 1-(β-aminophenethyl)-imidazole, (**1b**, X=NH_2_, see Figure 1) against a broad panel of pharmacological tests, they displayed excellent broad-spectrum *in vitro* antifungal activity. This observation gave rise to an extensive synthetic exploration to investigate the structure-activity-relationships (SAR) in this class of compounds [2]. The 2-(imidazolyl)-acetophenones, easily accessible from phenacylbromides and excess imidazole, were therefore very good starting materials. A wide variety of reactions were performed on these ketones ranging from reduction to the corresponding alcohols, followed by alkylations (**2**) (e.g., miconazole and analogues [3,4] see Scheme 1), addition reactions (**3**), thioether formation (**4**) (e.g., sulconazole), reductive aminations (**5**), and oxime formation (**6**) (e.g., oxiconazole [6]). Furthermore structurally related homologs to miconazole (**7**) were prepared from substituted phenacyl bromides or benzyl cyanides to gain more insight in the SAR of the series [5]. Butoconazole (**8**) is an outlier having a 1-(phenylbutyl)-imidazole scaffold [7] (Scheme 1). This extensive exploration has led to eight marketed compounds, of which miconazole was the first one (see Figure 2).

In the early days, profiling of compounds occurred via *in vitro* screening against a broad panel of yeast and moulds, followed by extensive *in vivo* screening of the most active compounds. The compounds **2a**-**2aa**, **3a**-**3c** and **5** were tested against the yeast *Candida albicans* (C.a), dermatophytes like *Microsporum canis* (M.c), *Trichophyton mentagrophytes* (T.m), *Trichophyton rubrum* (T.r), and the fungus *Aspergillus fumigatus* (A.f.) (see Table 1).

All the compounds in Table 1 displayed broad-spectrum activity; the least potent ones gave a partial inhibition of growth at 100 μg/mL against *C*. *albicans* with **2r **(R=H, X=O, Y=Z=H) as an example.

**Table 1:** *In vitro* antifungal activity of miconazole and analogues [2].

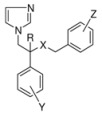									
	R	X	Y	Z	*C.a.^(a)^*	*M.c.*	*T.m.*	*T.r.*	*A.f.^(b)^*
**2a**	H	0	2,4-Cl_2_	2,4-Cl_2_	10	<1	0.1	<1	10
**2b**	H	0	2,4-Cl_2_	2,6-Cl_2_	100	<1	0.1	0.1	10
**2c**	H	0	4-F	2,4-Cl_2_	100	10	<1	<1	10
**2d**	H	0	2,4-Cl_2_	4-F	100	10	10	10	10
**2e**	H	0	2,4-Cl_2_	2-F	100	10	10	10	100
**2f**	H	0	2,4-Cl_2_	2-Cl	100	10	0.1	0.1	10
**2g**	H	0	4-Cl	2,6-Cl_2_	10	100	10	10	100
**2h**	H	0	2,4-Cl_2_	4-Cl	100	0.1	0.01	0.1	<1
**2i**	H	0	4-Cl	2,4-Cl_2_	10	10	<1	0.1	10
**2j**	H	0	2-Cl	2,4-Cl_2_	100	<1	<1	<1	10
**2k**	H	0	4-Br	2-Cl	100	10	10	10	100
**2l**	H	0	4-Br	4-Cl	10	<1	0.1	0.1	<1
**2m**	H	0	4-F	4-Cl	100	<1	<1	<1	10
**2n**	H	0	4-F	2-Cl	100	100	10	<1	100
**2o**	H	0	4-Cl	2-Cl	100	100	10	10	100
**2p**	H	0	4-Cl	4-Cl	10	<1	0.1	0.1	10
**2q**	H	0	H	4-Cl	100	10	<1	<1	10
**2r**	H	0	H	H	>100	100	10	10	100
**2s**	H	0	4-Me	2,4-Cl_2_	100	100	10	10	100
**2t**	H	0	2-Me	2,4-Cl_2_	100	10	10	<1	10
**2u**	H	0	2,4-Cl_2_	2-Me	100	10	0.1	0.1	10
**2v**	H	0	2-Me	2,4-Cl_2_	100	<1	<1	<1	<1
**2w**	H	0	2,4-Cl_2_	3-MeO	100	10	10	10	100
**2x**	H	0	2,4-Cl_2_	4-MeO	100	10	10	10	100
**2y**	H	0	4-Cl	2-Me	100	100	10	10	100
**2z**	H	0	4-Me	2-Cl	100	100	10	10	100
**2aa**	H	0	4-Me	4-Cl	100	10	<1	<1	10
**3a**	Me	0	4-Cl	2,4-Cl_2_	>100	10	10	10	100
**3b**	Me	0	H	2,4-Cl_2_	>100	100	10	10	>100
**3c**	Me	0	4-Cl	4-Cl	100	100	10	10	100
**5**	H	NH	4-Cl	2-Cl	>100	100	100	100	ND^(c)^
**tolnaftate**					>100	>100	10	<1	>100
**nystatin**					333	ND	ND	ND	ND

^(a)^ Lowest concentration of total inhibition (μg/mL); ^(b)^ *C.a.: Candida albicans; M.c.: Microsporum canis; T.m.: Trichophyton mentagrophytes;T.r.: Trichophyton rubrum; A.f.: Aspergillus fumigates* ^(b)^ Figures proceeded by "<" represent the lowest dose level tested (µg/mL) with complete inhibition; ^(c)^ Figures proceeded by ">" denoted partial growth at 100 µg/mL; **2a**: miconazole; **2b**: isoconazole; **2h**: econazole. ^(c)^ ND: not done.

The activity against *C*. *albicans* was considered as most essential because the few antimycotic agents available in that period displayed a low efficiency against infections caused by this agent. Substitution of both phenyl rings with a variety of substituents led to an increase in activity, but introduction of halogens in *ortho*- and *para*-position gave potent compounds like **2a** (R=H, X=O, Y=Z=2,4-Cl_2_), **2g** (R=H, X=O, Y=4-Cl, Z=2,6-Cl_2_), **2i **(R=H, X=O, Y=4-Cl, Z=2,4-Cl_2_), **2l** (R=H, X=O, Y=4-Br, Z=4-Cl), and **2p** (R=H, X=O, Y=Z=4-Cl) against *C*. *albicans* with complete inhibition of growth at 10 μg/mL. The compounds **2h **(R=H, X=O, Y=2,4-Cl_2_, Z=4-Cl), **2l** (R=H, X=O, Y=4-Br, Z=4-Cl), and **2v** (R=H, X=O, Y=2-Me, Z=2,4-Cl_2_), respectively displayed a remarkable activity against *A*. *fumigatus* with a complete inhibition of growth at 1 μg/mL and maybe lower. Against *T*. *mentagrophytes* compound **2h** was the most potent, with complete inhibition of growth at 0.01 μg/mL. Introduction of a methyl moiety in compound **2p** (R=H, X=O, Y=Z=4-Cl) resulted in compound **3c** (R=CH_3_, X=O, Y=Z=4-Cl), which gave a strong decrease in activity, not only against *C*. *albicans* but also against dermatophytes and *A*. *fumigatus*. Replacement of the oxygen in **2p** by an NH group (**5**) had a strong decreasing effect on activity against all the tested fungi. The best compounds were significantly more active than the reference compounds like tolnaftate and nystatin. In particular on behalf of the *in vitro* results against *C*. *albicans* it had been decided to investigate compound **2a** for further development and it received the generic name of miconazole. Compounds **2b** and **2h** were licensed out to the Schering (Bayer) and the Cilag company (Johnson & Johnson), respectively, and received the generic names isoconazole and econazole.

Structural modification of the miconazole structure in which the oxymethylene was replaced with a methyleneoxy or an ethyleneoxy moiety gave rise to a new series (see Table 2, **7a-c**) of compounds [5].

**Table 2:** *In vitro* and *in vivo* antifungal activity and *in vitro* antibacterial activity of some close miconazole analogues [5].

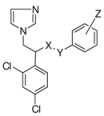												
Compound	X	Y	Z	M.c^.(a,b)^	T.m.	T.r.	Cr.n.	C.tr.^(c)^	C.a.	Muc.	A.f.	In vivo ^(d)^
**2a miconazole**	O	CH_2_	2,4-Cl_2_	1	<1	<1	1	100	10	>100	10	4/13
**7a**	CH_2_	O	2,4-Cl_2_	<1	<1	<1	<1	>100	>100	>100	>100	NT^(e)^
**7b**	(CH_2_)_2_	O	2,4-Cl_2_	10	<1	<1	<1	>100	100	100	100	2/2
**7c**	(CH_2_)_2_	O	4-F	10	<1	<1	<1	>100	10	100	100	2/2

^(a)^ Lowest concentration of total inhibition (μg/mL). *M.c.: Microsporum canis; T.m.: Trichophyton mentagrophytes; T.r.: Trichophyton rubrum; Cr.n.: Cryptococcus neoformans; C.tr.: Candida tropicalis; C.a.: Candida albicans;Muc.: Mucor species; A.f.: Aspergillus fumigatus;* ^(b)^ Figures proceeded by "<" represent the lowest dose level tested (µg/mL) with complete inhibition; ^(c)^ Figures proceeded by ">" denoted partial growth at 100 µg/mL; ^(d)^ Oral treatment (10 mg/kg) of cutaneous candidiasis by *C*.* albicans* in guinea pigs; Ratio of animals cured/infected ; ^(e)^ NT = not tested.

Compound **7a** (X=CH_2_, Y=O,) was equipotent or even more potent than miconazole against dermatophytes, but against *C*. *albicans* the compound displayed disappointing results and was *in vitro* clearly less active than miconazole. Based on *in vitro* experiments compound **7b **was equipotent to miconazole against dermatophytes like *T*. *mentagrophytes* and *T*. *rubrum*, but was clearly less active against *M*. *canis* and against the important yeast *C*. *albicans*. Oral treatment of cutaneous (10 mg/kg) candidiasis in guinea pigs with **7b **and miconazole indicated higher cure rates for **7b **[3]. For us these results were a first indication that *in vitro* and *in vivo* activity did not correlate very well.

Replacement of the 2,4-dichlorobenzyl group in the miconazole structure with a 2-(3-chloro-thienyl)-methyl moiety gave tioconazole **2ac **(Figure 2), a new broad-spectrum antifungal agent (see Figure 2) [17].

Structural modification on the econazole molecule by the substitution of a large phenylthio group for the 4-chloro in the benzylether moiety led to the synthesis of fenticonazole **2ab **(Figure 2), a compound with broad-spectrum activity (see Figure 2) [4,18,19,20,21,22].

Insertion of an oxime moiety into the miconazole structure gave oxiconazole **6 **(Figure 2), a new broad-spectrum antifungal agent [6]. The chemical structure contains an oxime-ether fragment, which can give rise to *E* and *Z* geometric isomers, of which the *Z*-form clearly demonstrated superior antifungal activity compared to the *E*-isomer.

Replacement of the oxygen by a sulfur in the econazole structure led to the discovery of sulconazole **4**, another broad-spectrum antimycotic agent developed for topical application (see Figure 2, Scheme 1) [23].

Substitution of the 2,4-dichlorobenzyl moiety in miconazole with a 3-(7-chlorobenzothiophene)-methyl group gave sertaconazole **2ad**, another imidazole derivative with broad-spectrum antifungal activity (see Figure 2) [24,25].

All the aforementioned imidazole derivatives are 1-(arylethyl)-imidazoles and elongation of the distance between the aryl and the imidazole rings gave rise to the discovery of the 1-(arylbutyl)-imidazole derivative butoconazole **8** (see Scheme 1) [7]. In an *in vitro* agar dilution assay the compound was highly active against dermatophytes, yeasts and Gram-positive bacteria. Butoconazole and its enantiomers gave similar *in vitro* activities against the *C*. *albicans* 523 strain [26].

**Figure 3:**
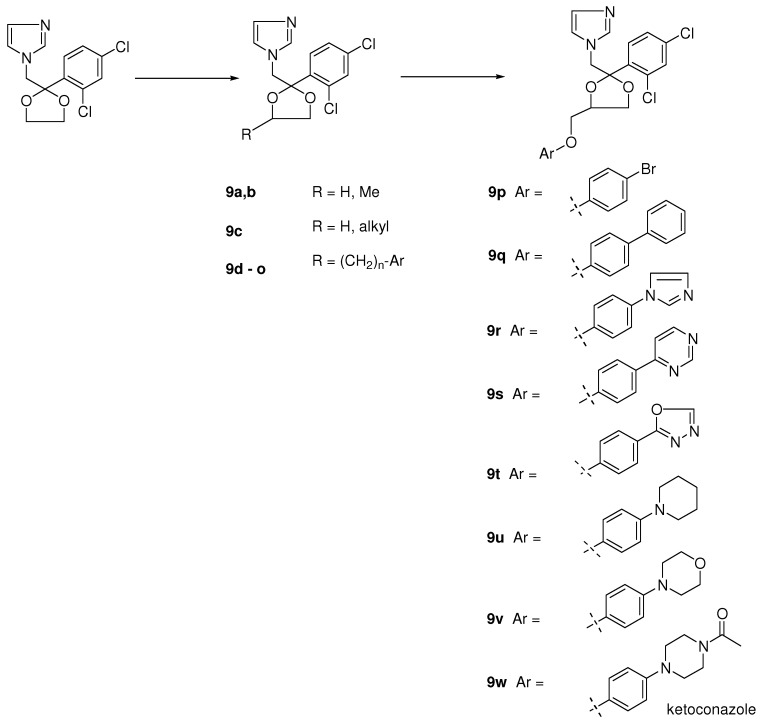
The evolution from simple imidazolyl dioxolanes to ketoconazole.

In the period when we synthesized miconazole and some analogues, we needed some dioxolane derivatives in order to protect the keto moiety in the phenacyl imidazoles (see Figure 3). To our big surprise the halogenated aryl derivatives displayed an interesting broad-spectrum antifungal activity [2]. We decided to synthesize more complicated dioxolanes, which were in fact new miconazole analogues containing an incorporated dioxolane ring (**9a-o**). Synthetic exploration of the dioxolane substitution eventually led to the discovery of ketoconazole as the first orally active broad-spectrum antifungal agent. Later in the process more potent topically active compounds like terconazole and the broad spectrum orally active antifungal agent itraconazole were discovered.

**Scheme 2:**
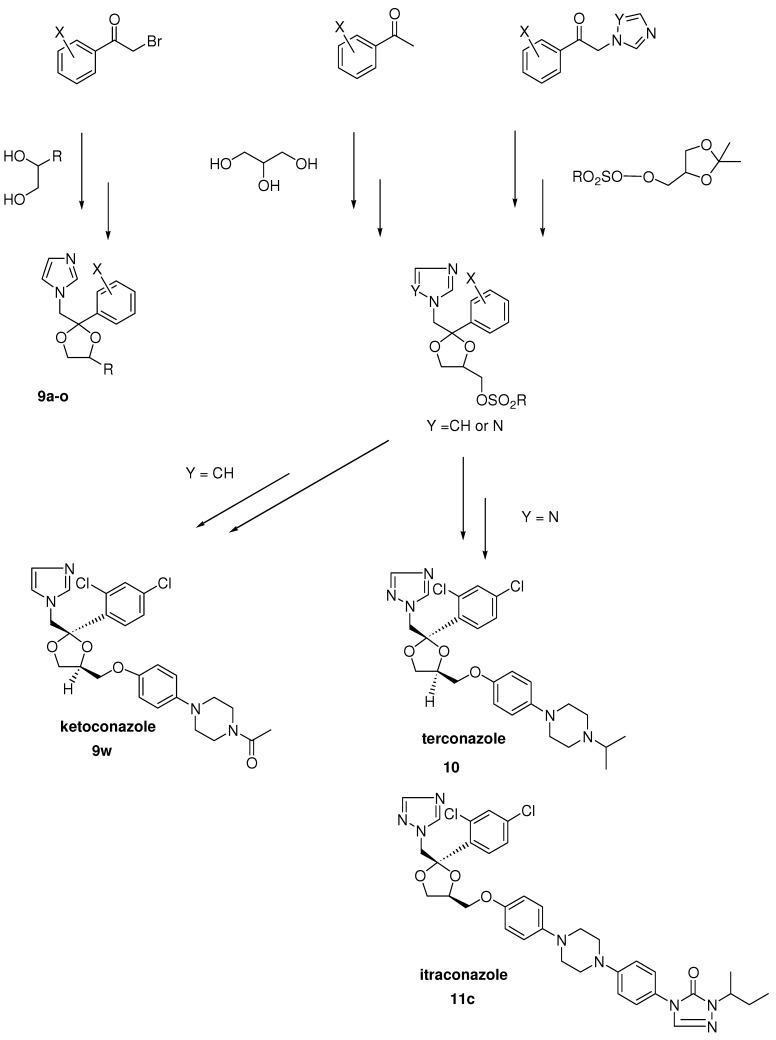
Synthesis of dioxolane derivatives of miconazole, and ketoconazole, terconazole and itraconazole.

Dioxolane analogues **9a-o** were prepared according to Scheme 2 [8]. Ketalization with diols was done in a mixture of benzene/*n*-butanol (stabilized aryl glycols) medium at reflux temperature with p-toluene sulphonic acid. Treatment with imidazole at high temperature in dimethyl formamide gave the compounds **9a-o **as *cis/trans* mixtures. Further exploration of the 1,3-dioxolane ring system was achieved via ketalisation using glycerol or trans ketalisation with sulphonated solketal. Nucleophilic substitution of the sulphonate by a variety of nucleophiles, and particularly phenols, eventually led to the discovery of ketoconazole, terconazole and itraconazole. The details for the synthetic procedures for ketalisation and synthesis of ketoconazole, terconazole en itraconazole have been described in the literature [8,9,10,11,12].

In general the compounds displayed very potent or marked *in vitro* antifungal activity against dermatophytes like *M*. *canis*, *T*. *mentagrophytes* and *T*. *rubrum* with activity ranging from < 1 to 10 µg/mL (see Table 3).

The miconazole analogue **9i** (X=Y=2,4-dichloro, n = 0) not only was very active against dermatophytes, but also surprisingly potent against *C*. *neoformans* and *A*. *fumigatus* at concentrations of lower than 1 µg/mL. On the other hand the *in vitro* activity against *C*. *albicans* was disappointing, as more than 100 µg/mL was needed to inhibit growth of the fungus. In contrast to the disappointing *in vitro* activity surprisingly high activity was observed in a rat vaginal candidiasis model where the two infected rats were cured after an oral dose of 10 mg/kg, which was clearly superior to oral treatment with miconazole resulting in none of the six treated animals cured at the same dose. This result and other *in vivo* results convinced us that it was difficult to draw conclusions from the *in vitro* data with regard to *C*. *albicans*. There was no correlation between *in vitro* and *in vivo* activity in the tests against *C*. *albicans* and in the different *Candida* animal models. Elongating the distance between the aryl group and the dioxolane ring in the 4-position with a methylene group resulted in compounds with a remarkable *in vitro* antifungal activity. Moreover the oral activity was markedly improved in induced skin candidiasis in the guinea pig by *C*. *albicans* compared to that of miconazole. However the activity in the rat vaginal candidiasis model remained disappointing [8]. The results were obtained for *cis*/*trans* mixtures because in that period it was too complicated or even impossible to separate the mixture.

**Table 3:** *In vitro* antifungal and antibacterial activities and *in vivo* antifungal data of miconazole analogues containing a dioxolane scaffold [8].

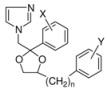														
	X	Y	n	M.c^.(a,b)^	T.m.	T.r.	Ph.v^.(c)^	Cr.n.	C.tr.	C.a.	Muc.	A.f.	Rat^(d,e)^	Guinea Pig ^(f)^
**9d**	4-Cl	2-Cl_2_	0	10	<1	<1	100	10	>100	>100	100	100	NT	NT
**9e**	4-Cl	2,4-Cl_2_	0	10	<1	<1	100	10	>100	100	100	100	1/2	2/2
**9f**	2,4-Cl_2_	H	0	<1	<1	<1	100	100	>100	100	100	<1	0/2	NT
**9g**	2,4-Cl_2_	2-Cl	0	10	<1	<1	100	<1	100	100	100	100	**-**	NT
**9h**	2,4-Cl_2_	4-Cl	0	<1	<1	<1	100	<1	10	100	10	<1	NT	1/2
**9i**	2,4-Cl_2_	2,4-Cl_2_	0	10	<1	<1	100	<1	>100	>100	10	<1	2/2	NT
**9j**	2,4-Cl_2_	4-Br	0	<1	<1	<1	100	<1	10	100	10	<1	0/2	3/4
**9k**	2,4-Cl_2_	H	1	<1	<1	<1	100	<1	10	100	10	<1	0/2	2/2
**9l**	2,4-Cl_2_	4-Cl	1	<1	<1	<1	100	1	10	10	10	10	0/2	2/2
**9m**	2,4-Cl_2_	4-Br	1	<1	<1	<1	100	<1	>100	10	<1	10	0/2	NT
**9n**	2,4-Cl_2_	4-Ph	1	10	<1	<1	>100	100	>100	>100	10	100	0/2	NT
**9o**	2,4-Cl_2_	4-Ph	2	<1	<1	<1	>100	10	>100	>100	>100	10	0/2	NT
**2a**	miconazole			1	<1	<1	100	1	100	10	>100	10	0/6	4/13

^(a)^ Lowest concentration of total inhibition (μg/mL); *M.c.: Microsporum canis; T.m.: Trichophyton mentagrophytes; T.r.: Trichophyton rubrum; Ph.v.: Phialophora verrucosum; Cr.n.: Cryptococcus neoformans; C.tr.: Candida tropicalis; C.a.: Candida albicans;Muc.: Mucor species; A.f.: Aspergillus fumigatus;* ^(b)^ Figures proceeded by "<" represent the lowest dose level tested (µg/mL) with complete inhibition; ^(c)^ Figures proceeded by ">" denoted partial growth at 100 µg/mL; ^(d)^ Oral treatment (10 mg/kg) of vaginal candidiasis by *C.albicans* in rats; Ratio of animals cured/infected; ^(e)^ NT = not tested; ^(f)^ Oral treatment (10 mg/kg) of cutaneous candidiasis by *C.albicans* in guinea pigs; Ratio of animals cured/infected.

Further modification led to the synthesis of 4-phenoxymethyl dioxolanes (Figure 3; **9p-w**). In this type of compounds we were able to do the separation into the respective *cis* and *trans* isomers.

The most interesting compounds were the 4-bromo- (X= Br, **9p**) and 4-phenyl (X= phenyl, **9q** ) analogues with a *cis* configuration. The 4-bromo compound **9p** was *in vitro* highly active against dermatophytes, *A*. *fumigatus* and other fungi and comparable with that of miconazole. Probably due to the low solubility into the medium the 4-phenyl analogue was only active against *C*. *neoformans* under the same test conditions. Against *C*. *albicans* both compounds unfortunately displayed no activity even at concentrations of 100 μg/mL. In contrast to the *in vitro* activity against *C*. *albicans*, both compounds demonstrated a convincing *in vivo* activity after oral treatment superior to that of miconazole in induced vaginal candidiasis in the rat and in experimental skin candidiasis in the guinea pig, again indicating that *in vitro* results were not consistent with *in vivo* ones. The corresponding *trans*-isomers were clearly less active [27].

Encouraged by the *in vivo* results after oral treatment we hoped that the introduction of a hetero-cyclic ring for the second phenyl would increase activity. Different heterocyclic rings were tried and among them the pyrimidine **9s**, imidazole **9r** and oxadiazole **9t** (see Figure 3) derivatives were the most interesting compounds. All three compounds were very potent after oral treatment of an experimental vaginal candidiasis in the rat, however only one compound (the imidazole derivative **9r**) displayed also activity after oral dosing in skin candidiasis in the guinea pig. Encouraging results were also obtained with the compound in other animal fungal infection models and the compound was considered as a potential clinical candidate. However in the toxicity tests the compound had an unacceptable profile and development of the compound was finished.

Notwithstanding this disappointing result we were convinced to be able to solve the current problems and decided to pursue the program. In a next step the aromatic heterocycles were replaced with saturated heterocyclic rings such as piperidine **9u**, morpholine **9v** and N-acetylpiperazine **9w**. The nitrogen in the piperazine derivative was protected with an acetyl moiety to simplify synthesis of the piperazine target compound. It appeared that the acetyl piperazine derivative was the most potent compound within the new series. Deacylation and introduction of other acyl groups revealed that the formyl and acetyl group delivered the most potent orally active agents within the new series with a preference for the acetyl moiety. This compound received the generic name ketoconazole **9w **(Scheme 2).

The compound displayed broad-spectrum *in vivo* activity in a wide range of experimental fungal infections caused by different fungi in a variety of animal models. Some other substituents instead of 2,4-dichloro on the phenyl in the 2-position of the dioxolane ring were introduced in order to evaluate substituent effects on activity. Only the 2,4-dibromo and the 2,3,4-trichloro analogues demonstrated an *in vivo* activity against an already established vaginal candidiasis infection in the rat and were comparable to ketoconazole. The corresponding triazole derivative of ketoconazole was prepared and was clearly less potent than ketoconazole in the *in vivo* tests [27]. As expected and again confirmed the *trans*-isomer of ketoconazole is less active than ketoconazole (*cis*-isomer).

In a prophylactic experiment (treatment starting the day of infection), a daily dose of 5 mg/kg gave 100% protection in vaginal candidiasis in rats, whereas with miconazole the same result was obtained with 80 mg/kg. Oral administration of ketoconazole prevented or cured crop candidiasis in turkeys, skin, systemic and deep candidiasis in guinea pigs, and vaginal candidiasis in rats [28]. The results gained from animal models were confirmed in clinical trials.

Oral ketoconazole is effective against superficial mycotic infections and in deep-seated fungal infections and it is the first broad-spectrum antifungal agent with oral activity. For all these indications the product is marketed in different countries.

Reduction of the acyl group in ketoconazole derivatives to alkyl groups led to the synthesis of a series of imidazole and triazole compounds with a marked topical activity against superficial fungal infections in animals, in particular vaginal candidiasis in rats. The oral activity of these compounds was lower than that of ketoconazole. Substitution of the imidazole ring with a triazole ring enhanced topical potency of the products. In this series terconazole **10** has been developed as a topical agent for the treatment of superficial fungal infections in humans (Table 4).

**Table 4:** *In vitro* antifungal activity of terconazole: Complete or Marked inhibition of growth at the concentrations indicated in Sabourand broth after 14 days of incubation of terconazole [10].

	Sabourand broth, inhibition (μg/mL)		Sabourand broth + 10% inact. Bovine serum, inhibition (μg/mL)	
Organism	Complete	Marked^(a)^	Complete	Marked^ (a)^
*Microsporum canus*	100	100	100	10
*Trichophyton mentagrophytes*	1	1	1	0.1
*Trichophyton rubrum*	100	10	10	1
*Cryptococcus neoformans*	10	1	1	0.1
*Candida albicans*	>100	10	100	0.1
*Candida tropicalis*	10	1	1	0.1
*Phialophora verrucosa*	100	10	10	1
*Sporothrix schenckii*	100	100	100	1
*Aspergillus fumigatus*	>100	>100	>100	100
*Mucor spp.*	>100	>100	100	100
*Saprolegnia spp.*	100	10	10	1

^(a)^ more than 50% inhibition of growth after 14 days of incubation.

**Figure 4:**
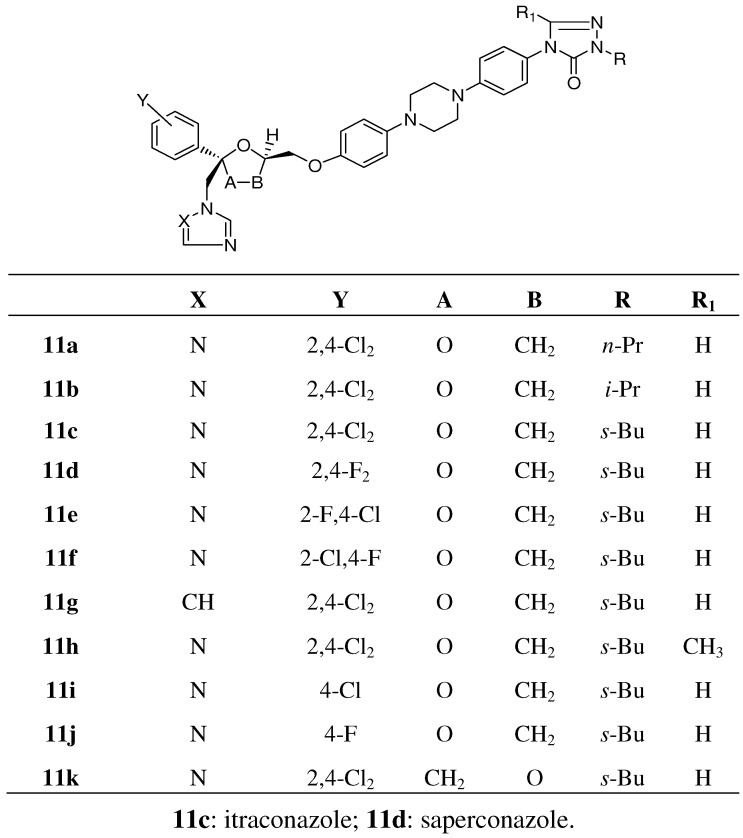
Analogues of itraconazole.

To our big surprise introduction of substituted aryl instead of alkyl groups gave no serious decrease in antifungal activity, indicating that many structural modifications were tolerated. In particular phenyl rings bearing substituted oxo-heterocycles in the para position of the phenyl ring generated compounds with superior oral antifungal activity. The most potent compounds were the aryl-triazolone compounds substituted with a *N*-1-branched alkyl group with *s*-butyl as an example, which received the generic name itraconazole **11c** and was highly orally active in experimental animal models like skin candidiasis and dermatophytosis in the guinea pig and vaginal candidiasis in the rat, respectively. Moreover the compound also was very potent in disseminated candidiasis and aspergillosis in the guinea pig. SAR studies in the itraconazole series revealed that the corresponding imidazole **11g** (see Figure 4) derivative of itraconazole displayed reduced activity compared to itraconazole.

Variation in the alkyl chain (R-group) of itraconazole (R=*s*-butyl) delivered compounds with decreased activity with an exception of the *iso*-propyl derivative **11b**, which demonstrated comparable or marginally lower oral antifungal activity in the different available animal models. The analogous n-propyl analogue **11a** was clearly less potent. Introduction of a methyl group (R’=methyl) in the 5-position of the triazolone ring **11h** had a negative effect on activity [11].

Replacement of the 2,4-dichloro substitution on the phenyl ring in the 2-position of the dioxolane ring by 4-chloro **11i**; 2-fluoro,4-chloro **11e**; 2-chloro, 4-fluoro **11f**; 4-fluoro **11j** or 2,4-difluoro **11d** (saperconazole) had a minor effect on *in vivo* activity [27].

An oxygen shift in the dioxolane (A-B=CH_2_O instead of OCH_2_) ring **11k** had a marginal effect on activity (Figure 4) [27]. In our case the SAR studies were based on oral and topical activity against vaginal candidiasis in the rat, infected with *C*. *albicans* and on induced skin dermatophytosis in the guinea pig caused by *M. canis*.

Further structure modification of the itraconazole structure finally led to the discovery of posaconazole by Schering-Plough. The enantioselective synthesis of posaconazole **12l** is described in the literature [13,14].

**Scheme 3:**
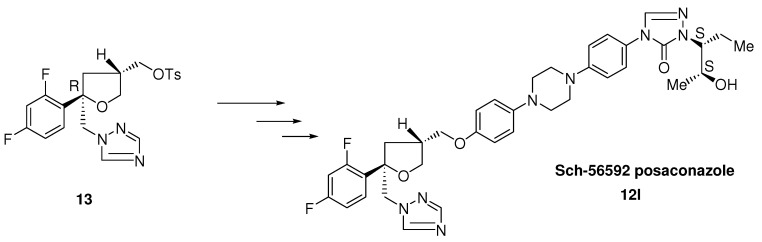
Synthesis of posaconazole from enantiomerically pure trisubstituted tetrahydrofurane.

**Figure 5:**
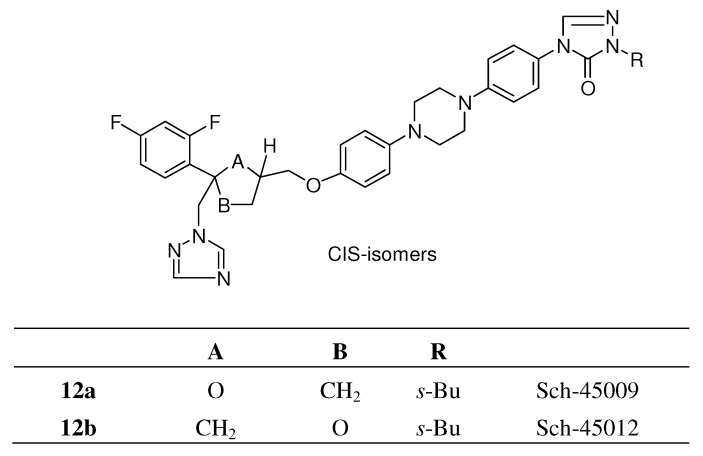
Tetrahydrofuryl isosters of itraconazole.

A team at Schering modified the 2,4 dichlorophenyl moiety of itraconazole into the 2,4-difluoro-phenyl group and the dioxolane ring into a tetrahydrofuran ring, resulting in two types of *cis*-isomers each consisting of a mixture of four stereoisomers (Figure 5) [14]. Both types had a comparable *in vitro* broad-spectrum activity. In contrast with *in vitro* activity the *in vivo* activity of Sch-45012 **12b** was clearly superior to that of Sch-45009 **12a** (see Figure 5) and itraconazole in systemic *Candida* and *Aspergillus* infection models. The corresponding *iso*-propyl analogue **12f** displayed a lower activity.

**Figure 6:**
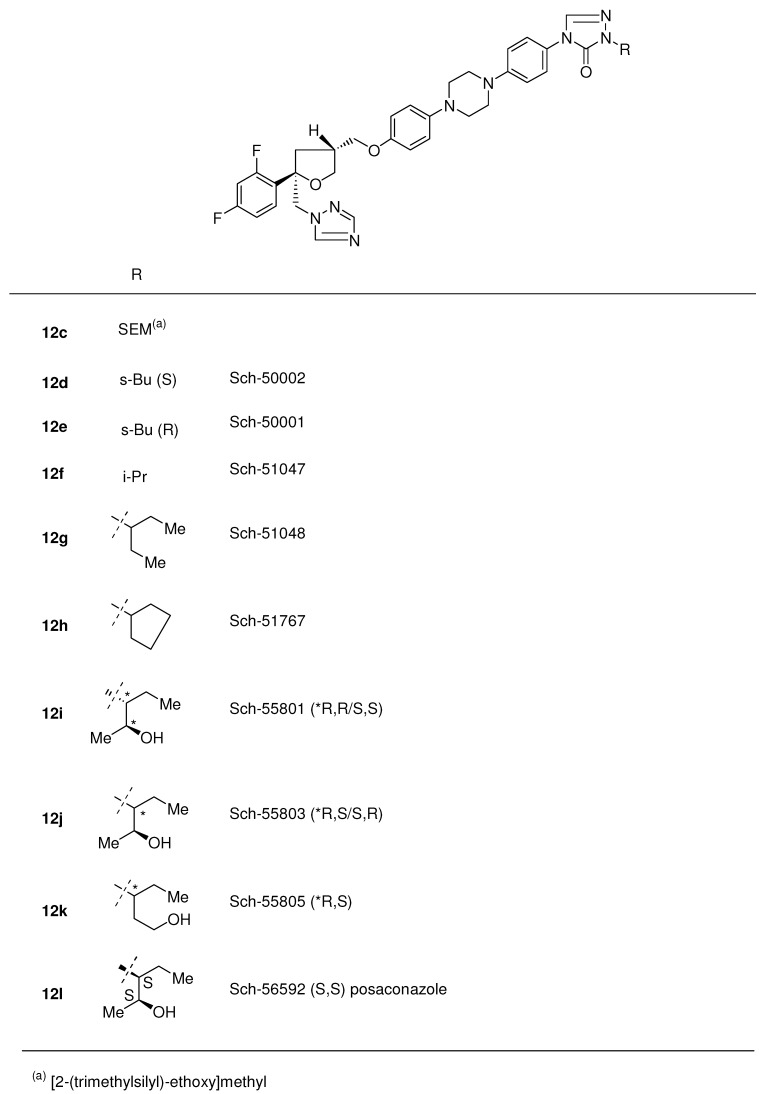
Posaconazole **12l** and analogues.

All four stereoisomers were primary evaluated for *in vitro* and *in vivo* activity. Results indicated that the two 5*S*-isomers Sch-49999 and Sch-50000 were virtually inactive and that the activity resided in the two 5*R*-enantiomers Sch-50001 **12e** and Sch-50002 **12d**. In this case there was an absolute stereochemical requirement for optimal oral activity in this series (Figure 6). Further attempts to eliminate chirality in the *s*-butyl group were unsuccessful but on the other hand the 3-pentyl analogue **12g** and one stereo centre less appeared to have improved therapeutic potential over Sch-50002 **12d** and other existing drugs against a variety of systemic fungal infections in normal and immuno-compromised infection models.

Notwithstanding the favourable oral activity of Sch-51048 **12g** an attempt was made to improve oral absorption and synthesis of analogues having polar side chains was undertaken. Introduction of these side chains learned that relatively basic or acidic side chains (Figure 6) resulted in reduced activity, whereas incorporation of hydroxylated side chains resulted in greatly improved activity. Preliminary *in vitro* evaluation of Sch-51048 hydroxylated analogues demonstrated that most analogues had excellent broad-spectrum activity against *C*. *albicans* (31 strains) and *A*. *fumigatus* (38 strains). Three analogues (Sch-56588, Sch-56592, and Sch-56984) derived from **12i** and **12j** showed the best overall biological profile and were subjected to detailed *in vivo* evaluation in several normal and immunocompromised infection models (*Candida* and *Aspergillus*). Based on overall efficacy and bioavailability in 5 animal species, Sch-56592 **12l** was recommended for further development [14] and is currently on the market and received the generic name posaconazole.

**Figure 7:**
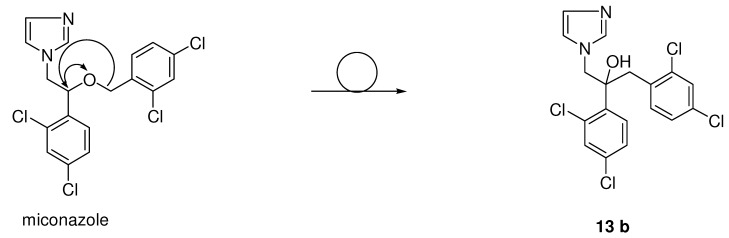
From miconazole to imidazolyl carbinols with antifungal activity.

**Scheme 4:**
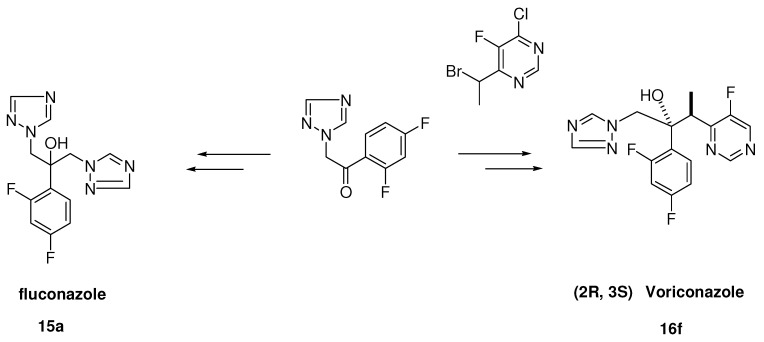
Preparation of fluconazole and voriconazole.

Another important series has been derived from the miconazole scaffold, namely the class of the azolyl-propanol-2 analogues were developed by Pfizer with fluconazole (**15a**) and voriconazole (**16f**) as examples (Scheme 4). Shifting the benzyl moiety in miconazole (see Figure 7) from the oxygen to the α-carbon and the hydrogen of the α-carbon to the oxygen within the phenethyl imidazole moiety gave rise to a member **13b** of a new series and type of antifungal agents, namely the 3-aryl-2-hydroxypropyl-azoles.

**Figure 8:**
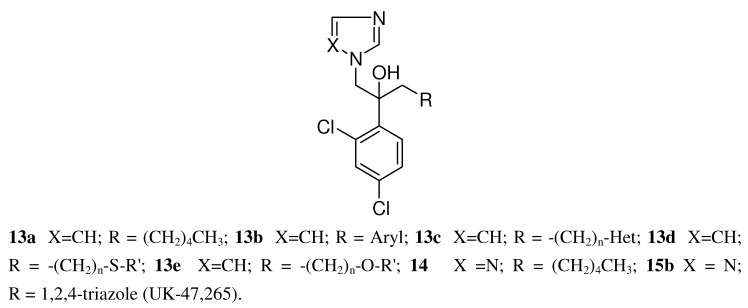
Structure of imidazolyl and 1,2,4-triazolyl carbinols.

Despite the preparation of almost 300 derivatives **13a**-**e** (see Figure 8) of this structural type a Pfizer team was unable to improve the *in vivo* efficacy of ketoconazole. In all these structural different compounds the imidazole ring was present and necessary for activity.

In the next steps there was a search for a metabolically more stable replacement for the imidazole ring in **13a** (R= pentyl) and the only one offering an encouraging improvement was the 1,2,4-triazole ring (see Figure 8). The resulting compound of type **14** (R= pentyl) was twice as potent *in vivo* as the corresponding imidazole derivative, despite being six times less potent *in vitro*. The metabolically vulnerable groups like alkyl or aryl finally were replaced with an 1,2,4-triazole ring bringing down lipophilicity and resulting in a symmetric triazole derivative **15b **(Table 5) with remarkable activity in the murine systemic candidiasis model.

The compound further displayed high *in vivo* activity in a broad range of systemic and superficial infection models, including infections caused by *Candida*, *Cryptococcus*, *Aspergillus* and dermatophytes. Unfortunately the compound appeared to be hepatotoxic in mice and in dogs and development of this compound was stopped.

In a follow-up program more than 100 triazoles were prepared with the focus on replacement of the 2,4-dichlorophenyl moiety. These compounds were tested orally in a systemic murine candidiasis model. The best compounds were further studied in mouse models of dermatophytosis and vaginal candidiasis. Aliphatic and heteroaromatic substituents were generally weakly active. The analogs containing a 2,4-dihalophenyl, a 4-halophenyl or 2,5-difluorophenyl group were very efficacious in these tests (see Table 5).

**Table 5:** Structures and *in vivo* activity of 1,3-bis-triazolyl propan-2-ols in the murine systemic candidiasis model [29].

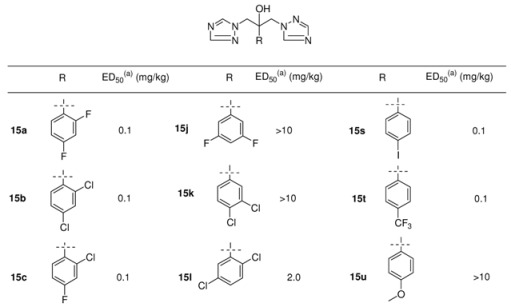
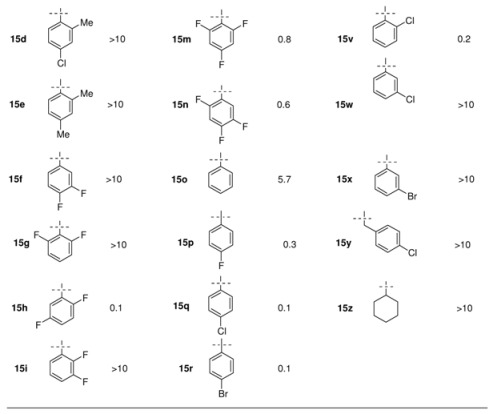
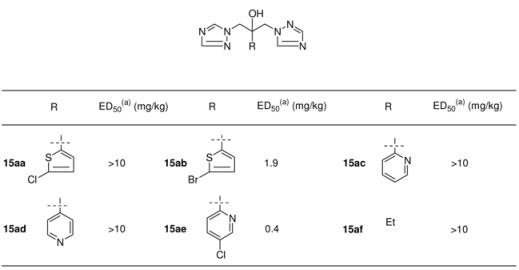

^(a)^ ED_50_: *in vivo* activity in a mouse model of systemic candidosis.

Pharmacokinetic evaluation in the mouse was clearly in favour of 2,4-difluorophenyl analogue **15a**, having a plasma half-life of 6.1 h and 75% of the drug was excreted unchanged in the urine [29]. Moreover the *in vivo* activity in different fungal mouse models was excellent and this compound was developed as an antifungal agent known under the generic name fluconazole.

Fluconazole is very successful in the treatment of infections due to *C*. *albicans* and *C*. *neoformans* but on the other hand it is poorly effective against *Aspergillus* infections when compared with itraconazole. Therefore it was of interest to find a next-generation antifungal agent, which would combine the favourable features of fluconazole with a broader spectrum of antifungal activity, including efficacy in aspergillosis.

As a first step a methyl group to the α-carbon of one of the triazole rings (see Table 6) was introduced **16a** which modification resulted in an increased potency against *A*. *fumigatus*. On the other hand the replacement of one of the triazoles in fluconazole with a 4-pyridyl moiety (**16b**, see Table 6) also gave a significant increase of *A*. *fumigatus* activity with an MIC of 3.15 μg/mL. Introduction of a methyl group on the a-carbon with regard to the pyridyl ring **16c** gave a further increase in activity. Mice infected with lethal inoculums of *A*. *fumigatus* survived after treatment with an oral dose of 20 mg/kg b.i.d. for 5 days. Unfortunately there were no cures.

**Table 6:** *In vitro* and *in vivo* activity of fluconazole and analogues against *A*. *fumigatus.*

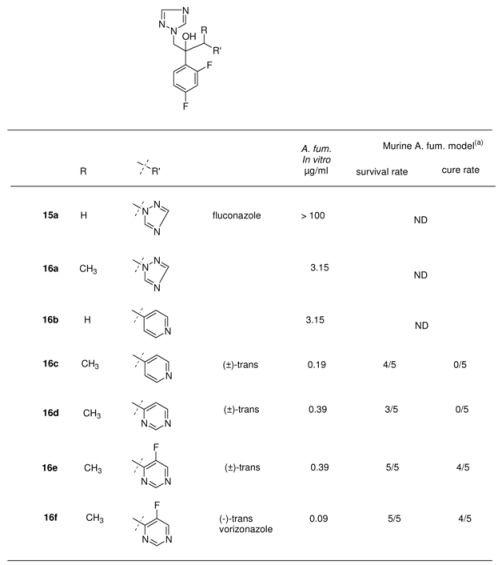

ND: no data; ^(a)^ *In vivo* activity in a murine (n = 5) *A. fumigatus* systemic model after oral treatment at 5 mg/kg b.i.d. for 5 days.

Introduction of another 6-membered heterocyclic ring such as unsubstituted pyrimidine **16d **and 5-fluoropyrimidin-4-yl ring **16e** led to a high activity against *A*. *fumigatus*, the 5-fluoro-4-pyrimidinyl **16e **derivative (*trans* isomer) being the most potent one with high cure rates. The compound was able to give high survival rates and cure rates in mice infected with *A*. *fumigatus* at a dose of 20 mg/kg twice daily for five days. The introduction of a fluorine in the pyrimidine ring was essential because animals treated with compounds **16c** and **16d** without a fluorine in the ring were able to survive but unfortunately no cures could be achieved in this group.

Based on the *in vitro* and *in vivo* activity and a good solubility profile it had been decided to further evaluate the 5-fluoro-4-pyrimidine compound. It appeared that the activity resided almost entirely in the (-)-enantiomer **16f** (voriconazole) (MIC 0.09 μg/mL), whereas the (+)-enantiomer inhibited the fungus at a concentration of 50 μg/mL. Compared with fluconazole the *in vitro* activity of voriconazole (**16f**) against *C*. *albicans*, *C*. *krusei*, *C*. *glabrata*, *C*. *neoformans*, and *A*. *fumigatus* was markedly improved [30].

For fluconazole the route described in Scheme 4 starts from the readily available precursor 2-triazolyl-2’,4’-difluoroacetophenone. Epoxidation of the ketone followed by ring opening with 1,2,4-triazole of the epoxide finally gives target product fluconazole **15a **[15]. The synthetic route leading to voriconazole (Scheme 4) is rather complicated, because the molecule contains two asymmetric centres. The absolute configuration of voriconazole is 2*R*,3*S*. A highly efficient route to the key intermediate 4-chloro-5-fluoro-6-ethyl-pyrimidine has been described in the literature [16]. Alpha bromination of the ethyl substituent with *N*-bromosuccinimide and azoisobutyronitrile in refluxing dichloromethane gave the 6-(1-bromoetyl)-pyrimidine in high yield. This compound was coupled diastereoselectively under Reformatsky reaction conditions with zinc powder and iodine in tetrahydrofuran with 1-(2,4-difluorophenyl)-2-(1*H*-1,2,4-triazol-1-yl)-1-ethanone giving a 65/35 ratio of diastrereomeric pairs of the adduct, which were separated via flash chromatography into *RR/SS*- and *RS/SR*-pairs. In the most active forms there was a *RS/SR*-orientation between the methyl- and the hydroxy group.

The *RS/SR-*compound as a free base was dehalogenated with 5% w/w palladium-on-carbon via hydrogenation. Voriconazole (**16f**) was prepared from the racemate via a diastereomeric salt resolution process using (1R)-10-camphorsulfonic acid [16]. Finally the target compound was isolated as a hydrogen chloride salt.

## 3. Pharmacology and Clinical Results

### 3.1. Topical agents

#### 3.1.1. Miconazole (**2a**, Figure 2)

In the sixties a limited number of compounds like undecylenates, diamthazole and tolnaftate were available for topical treatment of dermatophyte infections and for oral treatment only griseofulvin was available. Treatment of yeast infections, and in particular *Candida* infections, were based on antibiotic polyenes like nystatin, pimaricin and amphotericin B, all of which had a relatively high toxicity. Amphotericin B was available for intravenous applications against a wide variety of systemic life-threatening fungal infections and had to be used carefully. These compounds were ineffective or only slightly effective against dermatophyte infections, so the need for broad-spectrum and safer compounds was acute.

In contrast to the limited activity of undecylenates, diamthazol, nystatin, and tolnaftate, miconazole has a broad-spectrum activity against most pathogenic fungi, such as dermatophytes and *Candida* and against numerous saprophytic fungi. Moreover the compound displayed activity against Gram-positive bacteria, however no activity was found against Gram-negative bacteria [2,31].

Miconazole is very active against dermatophytes, especially against *Trichophyton mentagrophytes*, *T*. *rubrum* and *Epidermophyton floccosum* (Table 7). In the majority of the dermatophytes studied, growth was completely inhibited at 1 μg/mL. Against some species a marked fungistatic activity was observed at concentrations as low as 0.01 μg/mL [32].

**Table 7:** MIC’s (μg/mL) of sertaconazole, fluconazole, miconazole, econazole, isoconazole, sulconazole, butoconazole, ketoconazole, fenticonazole, and itraconazole against clinical isolates of different fungal species.

Strains testesd	Number of isolates	Antifungal Agent	MIC range	MIC_90_	Ref.
*Trichophyton*	7	Fenticonazole	0.28 – 20		[20,21]
*mentagrophytes*	2	Miconazole	0.312 – 80		[20,21]
	2	Econazole	0.60 – 0.80		[20]
*Trichophyton*	2	Fenticonazole	0.33 – 0.625		[20,21]
*rubrum*	2	Miconazole	0.312 – 0.55		[20,21]
	1	Econazole	0.58		[20]
*Trichophyton*	1	Fenticonazole	1.25		[21]
*verrucosum*	1	Miconazole	10		[21]
*Trichophyton*	1	Fenticonazole	0.25		[20]
*tonsurans*	1	Miconazole	8		[20]
	1	Econazole	4		[20]
*Microsporum*	2	Fenticonazole	5 – 20		[21]
*Canis*					
*Microsporum*	2	Fenticonazole	8 – 9.2		[20]
*audouini*	2	Miconazole	9 – 10		[20]
	2	Econazole	10		[20]
*Microsporum*	2	Fenticonazole	5 – 8.8		[20,21]
*gypseum*	1	Miconazole	10 – 12		[20,21]
	1	Econazole	10		[20]
*Epidermophyton*	2	Fenticonazole	0.625 – 1.1		[20,21]
*floccosum*	1	Miconazole	0.4 – 0.625		[20,21]
	1	Econazole	1.3		[20]
*Candida*	106	Fenticonazole	0.25 – 32	8^(a)^	[33]
*albicans*	106	Miconazole	0.25 – 32	16	[33]
	106	Econazole	0.25 – 32	16	[33]
	106	Sulconazole	0.12 – 8	1	[33]
	106	Butoconazole	0.12 – 8	1	[33]
	106	Isoconazole	0.12 – 4	4	[33]
	30	Ketoconazole	0.03 – 0.25	0.5^(b)^	[25]
	30	Fluconazole	0.5 – 64	4	[25]
	30	Sertaconazole	0.03 – 1	0.06	[25]
	30	Itraconazole	0.03 – 0.125	0.03	[25]
*Candida*	24	Fenticonazole	0.25 – 1	0.5^(a)^	[33]
*parapsilosis*	24	Miconazole	0.12 – 0.5	0.5	[33]
	24	Econazole	0.25 – 4	4	[33]
	24	Sulconazole	0.12 – 8	2	[33]
	24	Butoconazole	0.12 – 1	0.25	[33]
	24	Isoconazole	0.12 – 0.25	0.25	[33]
	18	Ketoconazole	0.03 – 0.06	0.06^(b)^	[25]
	18	Fluconazole	1 – 8	8	[25]
	18	Sertaconazole	0.06 – 0.5	0.25	[25]
	18	Itraconazole	0.03 – 0.06	0.06	[25]
*Candida*	19	Fenticonazole	0.5 – 16	8^(a)^	[33]
*tropicalis*	19	Miconazole	0.5 – 16	8	[33]
	19	Econazole	0.5 – 16	8	[33]
	19	Sulconazole	0.25 – 8	4	[33]
	19	Butoconazole	0.12 – 1	1	[33]
	19	Isoconazole	0.12 – 2	2	[33]
	20	Ketoconazole	0.03 – 4	4^(b)^	[25]
	20	Fluconazole	4 – 64	64	[25]
	20	Sertaconazole	0.125 – 8	2	
	20	Itraconazole	0.03 – 64	64	
*Candida krusei*	9	Fenticonazole	4 – 16	16^(a)^	[33]
	9	Miconazole	2 – 4	4	[33]
	9	Econazole	4 – 8	8	[33]
	9	Sulconazole	4 – 8	8	[33]
	9	Butoconazole	0.25 – 0.5	0.5	[33]
	9	Isoconazole	1 – 2	2	[33]
	15	Ketoconazole	0.03 – 2	0.5^(b)^	[25]
	15	Fluconazole	4 – 64	64	[25]
	15	Sertaconazole	0.06 – 4	1	[25]
	15	Itraconazole	0.03 – 64	64	[25]
*Candida*	20	Fenticonazole	2 – 8	4^(a)^	[33]
*guilliermondii*	20	Miconazole	0.25 – 1	1	[33]
	20	Econazole	0.5 – 4	4	[33]
	20	Sulconazole	0.5 – 2	2	[33]
	20	Butoconazole	0.12 – 0.25	0.12	[33]
	20	Isoconazole	0.12 – 1	0.5	[33]
	20	Ketoconazole	0.5 – 4	1	[33]
*Candida*	11	Ketoconazole	0.03 – 2	2^(b)^	[25]
*glabrata*	11	Fluconazole	1 – 64	64	[25]
	11	Fenticonazole	0.5 – 1	1	[25]
	11	Sertaconazole	0.125 – 0.5	0.25	[25]
	11	Itraconazole	0.03 – 64	16	[25]
	84	Miconazole	< 0.05 – 3.125	1.56^(c)^	[144
	84	Tioconazole	< 0.05 – 12.5	0.2	[144]
	84	Econazole	< 0.05 – 12.5	1.56	[144]
*Cryptococcus*	3	Fenticonazole	0.312 – 0.55		[20,21]
*neoformans*	2	Miconazole	0.625 – 1.95		[20,21]
	2	Econazole	2.25 – 2.62		[20]
*Geotrichum*	1	Fenticonazole	40		[20]
*candidum*	1	Miconazole	40		[20]
	1	Econazole	40		[20]
*Torulopsis*	1	Fenticonazole	40		[20]
*glabrata*	1	Miconazole	1.25		[20]
	1	Econazole	0.31		[20]
*Malassezia*	12	Ketoconazole	0.11 – 3.12	2.9	[73]
*furfur*	12	Econazole	0.78 – 6.25	4.8	[73]
	12	Miconazole	0.88 – 6.25	5.2	[73]
	12	Itraconazole	0.05 – 3.12	2.7	[73]
	12	Fluconazole	0.07 – 25	4.8	[73]
*Aspergillus*	2	Fenticonazole	22.3 – 160		[20,21]
*fumigatus*	1	Miconazole	33.1 – 160		[20,21]
		Econazole	28		[20]
*Aspergillus*	2	Fenticonazole	10 –37.5		[20,21]
*niger*	2	Miconazole	22 – 40		[20]
	1	Econazole	18		[20]
*Aspergillus*	1	Fenticonazole	10		[20]
*flavus*	1	Miconazole	12		[20]
	1	Econazole	2		[20]

^(a)^agar dilution plate method, ^(b)^ Casitone medium, ^(c)^ yeast nitrogen base medium.

The most sensitive yeast species like *Cryptococcus neoformans*, *Pityrosporum pachydermitis*, *P*. *orbiculare* and *Trichosporum cutaneum* were completely inhibited at 1 μg/mL. No growth of *C*. *albicans* was observed at 10 μg/mL, whereas there was a limited effect on growth at a concentration of 1 μg/mL. On a comparative basis miconazole was clearly more active than nystatin against all the yeasts tested and especially against *C*. *neoformans* [31].

Against different *Candida* species, including a large panel of 186 pathogenic *Candida* isolates miconazole was highly active with activity ranging from 0.12 to 32 μg/mL (Table 7) [31,33]. Miconazole seems to suppress adenosine-5’-triphosphate levels (ATP) in *C*. *albicans* cells [34]. Moreover miconazole displayed pronounced activity against dimorphic fungi like *Histoplasma capsulatum*, *Blastomyces brasiliensis* and *B*. *dermatitidis*, which were tested in the mycelium phase of the three dimorphic fungi with an activity ranging from 0.1 to 0.001 μg/mL [31].

The *Aspergilli* proved to be almost completely inhibited at a concentration of 10 μg/mL. Other fungi like *Sporothrix schenckii*, *Cladosporum werneckii*, *C*. *trichoides* and *Phialophora pedrosi* were completely inhibited at 1 μg/mL. The addition of bovine serum to the medium had an inhibitory effect on the *in vitro* antifungal activity of miconazole [31].

Prophylactic topical treatment with 2 and 0.5% miconazole ointment completely cured guinea pig skin infection caused by *T*. *mentagrophytes* (treatment starting the day of infection). Similar results were obtained for skin infection induced by *M*. *canis* with 2% miconazole. When treatment started after 3 days (therapeutic treatment) following the infection miconazole was highly effective against skin infections caused by *T*. *mentagrophytes* but to a lesser extent to infections caused by *M*. *canis*. The results obtained with tolnaftate were similar in these models at the same concentrations.

Oral treatment with 160 mg/kg of miconazole for 14 days was effective against skin dermatophytoses caused by *T*. *mentagrophytes* and *M*. *canis*, whereas a lower oral dose of 40 mg/kg was weakly active or not active at all. Topical treatment with 2% miconazole ointment of skin candidiasis caused by *C*. *albicans* in guinea pigs responded very well.

Oral treatment with 160 and 40 mg/kg gave very encouraging results, nearly all animals were cured, but a slight to marginal effect was observed at 10 mg/kg [31]. All acute toxicity studies as well as chronic toxicity studies demonstrated that miconazole was well tolerated [31].

Miconazole has a low bioavailability in man, which was associated with low plasma concentrations after oral administration [32]. The majority of the dose could be recovered from the faeces as unmetabolized drug. Acceptable plasma concentration of miconazole could be achieved after intravenous administration [36].

In humans topical treatment of fungal skin infections, caused by different pathogenic fungi gave high cure rates [37] with miconazole. Moderate results were obtained in onychomycosis and in infections of the scalp. Miconazole gave favourable results in vaginal candidiasis, in general cure rates of around 90% were obtained, even in patients with predisposing conditions.

Encouraging results with intravenous miconazole were observed in life-threatening mycoses like coccidiodomycosis, cryptococcosis, histoplasmosis, and paracoccidiodomycosis and in many cases were life-saving [37].

#### 3.1.2. Econazole (**2h**, Figure 2)

Econazole demonstrated similar *in vitro* activity against a wide range of fungi (Table 7), and several Gram-positive bacteria. Against dermatophytes, econazole in general was more potent than the reference compound tolnaftate, the MIC for total inhibition of *Microsporum* species ranged from 0.1 to 1.0 μg/mL and for *Trichophyton* species from 0.01 to 1.0 μg/mL. Moreover econazole was clearly more active than nystatin against yeasts like *T*. *glabrata* and *C*. *tropicalis* with MIC’s for total inhibition of 100 μg/mL [2,41,43]. In particular the difference in potency was pronounced against *C*. *neoformans*, *C*. *tropicalis*, *C*. *stellatoidea* and *C*. *parapsilosis* with MIC’s of 10 μg/mL [38]. Against *S*. *cerevisiae*, *C*. *albicans* and *C*. *krusei* both compounds were equipotent with MIC’s of 100 μg/mL [38]. In another *in vitro* study the compound was tested against a large panel of 186 pathogenic *Candida* clinical isolates with MIC’s ranging from 0.25 to 32.0 μg/mL [33].

Furthermore the compound demonstrated activity against other fungi like *A*. *niger*, *Madurella grisea*, *M. mycetoma*, *S*. *schenckii*, *B*. *dermatitidis*, *B*. *brasiliensis* and *H. capsulatum* [38]. Econazole had a similar activity against Gram-positive bacteria as miconazole and displayed no activity against Gram-negative bacteria [38].

Oral treatment against cutaneous dermatophytosis in guinea pigs caused by *T*. *mentagrophytes* and *M*. *canis* was only effective at high doses of 160 mg/kg administrated for 14 days and curing all treated animals, whereas 40 mg/kg was clearly less effective [38].

Oral econazole at doses of 160 mg/kg daily was effective in skin candidiasis in guinea pigs and in vaginal candidiasis in rats [38]. Intramuscularly econazole and miconazole administrated twice daily protected mice exposed to a lethal intranasal dose of *Coccidioides immitis* arthrospores at doses of 36 to 72 mg/kg [39].

Topical treatment of skin candidiasis in guinea pigs revealed that 2% econazole ointment was superior to 2% nystatin or 2% amphotericin B. In the prophylactic treatment of skin dermatophytosis caused by *T*. *mentagrophytes* in guinea pigs, econazole and tolnaftate applied as 2 and 0.5% ointments displayed similar activity. In *M*. *canis* infections econazole was slightly more active.

In curative tests econazole was active against both organisms, which resulted in disappearance of most lesions at day 42 of treatment [38].

A limited study performed in healthy volunteers demonstrated that oral absorption of econazole was disappointingly low [40]. Topical treatment of vaginal candidiasis gave high cure rates of 85 to 90% in patients after one treatment course and the response in vaginal candidiasis did not appear to be altered by the use of oral contraceptives [41].

In common dermatomycosis the overall clinical cure rate after treatment with econazole was about 90% [41]. In patients with otomycosis due to *Aspergillus* or *Candida* species, a 1% lotion of econazole gave high cures after 4 days to three weeks of treatment [41].

Patients with chronic or severe seborrheic dermatitis of the scalp often accompanied by severe pruritis and resistant to previous treatments could be successfully treated with econazole spray solution twice daily for up to 12 weeks [42].

#### 3.1.3. Isoconazole (**2b**, Figure 2)

Isoconazole has a broad-spectrum *in vitro* activity against dermatophytes, pathogenic yeasts, filamentous fungi, and Gram-positive bacteria [43]. In another *in vitro* study the compound was tested against a large panel of 186 pathogenic *Candida* clinical isolates with the MIC’s ranging from 0.12 to 4.0 μg/mL (Table 7) [33].

In animal models of fungal infections orally applied isoconazole gave negative results [44]. Initially isoconazole has been developed as a-once-a day, topical anti-*Candida* agent for the treatment of vaginal candidiasis. Clinical studies made clear, that after a single vaginal application of a 600 mg dose very little of the drug entered the blood. Evaluation studies with isoconazole have demonstrated that 80 to 90% of patients with vaginal candidiasis who were treated once a day with the drug remained clinically and mycologically cured [44,45].

Insertion of two 300 mg tablets delivered concentrations of isoconazole in the vagina, which remained above minimum inhibitory and fungicidal levels for at least 72 h [44].

Topical treatment with isoconazole of patients with dermatomycoses caused by dermatophytes like *Microsporum* species, *Trichophyton* species, *Epidermophyton* species, the yeasts *C. albicans*, *Candida* species, *Pityrosporum furfur* and other fungi gave cure rates ranging from 96 to 100% [46]. In another clinical study the 1% cream formulation was very effective in dermatomycosis. In patients with markedly inflammatory and eczematous mycoses addition of 0.1% of diflucortolone-21-valerate to the formulation was beneficial [47].

Coal miners with mycotic toenail and foot infections due to *Hendersonula toruloida*, *Scytalidium hyalinum* and dermatophytes like *T*. *rubrum*, *T*. *mentagrophytes* and *E*. *floccosum* were treated with 1% cream of isoconazole nitrate, giving clinical cure rates six weeks after treatment of 80% [48].

#### 3.1.4. Fenticonazole (**2ab**, Figure 2)

In acute and chronic toxicity studies fenticonazole appeared to be well tolerated in different animal species [19]. The compound demonstrated potent *in vitro* activity, ranging from 0.3–20 μg/mL [18] against dermatophytes such as the different *Microsporum* and *Trichophyton* species and *E*. *floccosum* and was comparable with that of miconazole and clotrimazole.

High activity was observed against *C*. *neoformans*, with an MIC of 0.31 μg/mL, which was in contrast with the moderate activity against *C*. *albicans* strains with an MIC of 20 μg/mL [18]. In another *in vitro* study the compound was tested against a large panel of 186 pathogenic *Candida* clinical isolates with the MICs ranging from 0.12 to 32.0 μg/mL (Table 7) [33]. The lowest concentration to inhibit *S*. *schenckii* and *A*. *niger* was 2.5 and 10 μg/mL, respectively.

High concentrations of 160 μg/mL were needed to inhibit *A*. *fumigatus* [18]. Potent antibacterial activity was demonstrated against Gram-positive bacteria like *S*. *aureus* and *S*. *pyogenis* strains and *Bacillus subtilis* with MIC’s ranging from 0.009 to 0.156 μg/mL, In another study the *in vitro* activity was evaluated against yeasts with variation of the pH of the medium. Fenticonazole and the reference compound miconazole both were highly active within a pH range of 4-5 [19,20].

In an experimental skin infection model in guinea pigs caused by *M*. *canis* both fenticonazole and miconazole demonstrated comparable activity after topical treatment [21]. Fenticonazole gave good results in skin candidiasis caused by *C*. *albicans* in guinea pigs [21,22].

Topical treatment with fenticonazole as a 2% cream or 100 mg ovules gave high cure rates in patients with vaginal candidiasis [44]. In patients with dermatomycoses or tinea versicolor high cure rates were obtained after topical treatment with fenticonazole 2% cream [44].

In numerous clinical studies, in part double blind fenticonazole gave a favourable clinical outcome and comparable to the results obtained with reference compounds like econazole, miconazole and clotrimazole [49], respectively.

#### 3.1.5. Oxiconazole (**6**, Figure 2)

Oxiconazole was highly active *in vitro* against dermatophytes like different *T*. *mentagrophytes*, *T*. *rubrum*, *M*. *canis*, *M*. *gypseum* and *E*. *floccosum* strains with an MIC ranging from 0.03 to 3 μg/mL. Moreover against different strains of *C*. *albicans*, *C*. *tropicalis*, *C*. *krusei*, *C*. *guillermondii*, *C*. *parapsilosis*, *Torulopsis glabrata* and *C*. *neoformans* the compound gave MIC values ranging from 0.001 to 100 μg/mL (Table 8) [50,51]. Furthermore strains of the moulds *A*. *fumigatus*, *A*. *niger*, *A*. *nidulans* and *A*. *flavus*, *Rhizopus rhizopodiformis*, *Absidia corumbifera* and *Mucor circinelloides* were susceptible to oxiconazole with MIC’s between concentrations of 0.1 and 100 μg/mL. Dematiaceous fungi were very sensitive to oxiconazole, exemplified by *Fonsecae pedrosi*, *Cladosporum trichoides* and *Madurella mycetoma* with MIC’s ranging from 0.001 to 0.1 μg/mL. Against biphasic fungi such as *H*. *capsulatum* and *C*. *immitis* oxiconazole gave MIC’s of 0.1 and 0.2 μg/mL, respectively [50].

**Table 8:** *In vitro* antifungal activity of oxiconazole [51].

Fungi	Number tested	MIC_90_	Range
**Pathogenic yeasts**	47		
*Candida albicans*	17	128	0.12 – 128
*Candida tropicalis*	9	32	0.06 – 32
*Candida parapsilosis*	6	4	0.06 – 64
*Cryptococcus neoformans*	10	0.13	0.06 – 0.25
*Torulopsis glabrata*	10	0.50	0.25 – 0.50
**Filamentous fungi**	81		
*Aspergillus flavus*	10	1	0.25 – 2
*Aspergillus fumigatus*	10	2	0.50 – 4
*Mucor*species	5	4	0.1 – 4
*Pseudallescheria boydii*	1	32^a^	
*Sporothrix schenckii*	9	1	0.50 – 4
Dermatophytes	29	1	0.06 – 4

MIC_90 _= concentration (μg/mL) which inhibits 90% of isolates tested, ^a ^MIC.

Oxiconazole was tested in Yeast Nitrogen Base and in Casitone liquid medium against 65 *C.* *albicans* strains, resulting in a different outcome for the MIC’s ranging from < 0.1–12.5 and 0.4–50 μg/mL, respectively. These experiments made clear that the antifungal MIC of an azole compound can vary and depend on the test method (inoculum size) and can further depend on the composition and pH of the medium [50,51].

In an *in vivo* experiment a topically applied 0.2% ointment of oxiconazole was highly effective in a trichophytosis guinea pig model with a complete protection of all treated animals [50]. At a much lower concentration of 0.05% only 50% of the animals remained free of mycosis.

Topical treatment starting 24 h post-infection of vaginal candidiasis in the rat with oxiconazole in a concentration range of the drug (0.03 to 1%) gave a significant reduction in the number of *C*. *albicans* colonies grown from the vaginal smears. The oral activity of oxiconazole in the treatment of systemic murine mycoses was weak [50].

Systemic absorption after topical application of ^14^C-labeled oxiconazole in volunteers was low, the majority of the dose was being found in the stratum corneum. [52].

In tinea pedis infections including *T*. *rubrum* and *T*. *mentagrophytes* as causative agents 82 and 83% cure rates were obtained after once or twice daily treatment with a 1% oxiconazole cream [52]. Similar results were observed in another clinical study and in both studies the cream was well tolerated.

Patients affected with tinea corporis and tinea cruris caused by *T*. *rubrum*, *T*. *tonsurans*, *M*. *canis*, *T*. *mentagrophytes* and *E*. *floccosum*, respectively were successfully treated with oxiconazole cream, applied once or twice daily with cure rates of 83% [52].

The treatment of patients with tinea versicolor caused by *M*. *furfur* gave cure rates of 83 and 82% after once or twice daily treatment. In cutaneus candidiasis encouraging results were obtained. Once or twice daily application of oxiconazole cream gave similar clinical results [53]. High cure rates of 92% were obtained with single dose oxiconazole (600 mg vaginal tablet) in patients with vaginal candidiasis [44].

#### 3.1.6. Tioconazole (**2ac**, Figure 2)

Tioconazole is a broad-spectrum antimycotic agent with *in vitro* activity against dermatophytes, yeasts and moulds. Against dermatophyte strains derived from *M*. *canis*, *M*. *gypseum*, *T*. *rubrum* and *T*. *mentagrophytes* the MIC ranged from 0.08 to 0.5 μg/mL. Different strains of *C*. *albicans*, *Candida* spp., *C*. *neoformans* and *T*. *glabrata* were susceptible with MIC’s varying from 0.05 to 12.5 μg/mL. Moreover the activity against *A*. *fumigatus* strains and *Aspergillus* species gave MIC’s in the range of 3.1 to 9.4 μg/mL [17,55].

Tioconazole behaves like other antimycotic azoles; there was an influence of the growth medium on antifungal activity [22]. Tioconazole has been reported to be more active than miconazole [17] but in another study both compounds appeared to be essentially equally active [54].

In mice infected via intravenous administration of a *C*. *albicans* suspension both tioconazole and miconazole when administered orally, subcutaneously, or intravenously significantly reduced the number of viable *C*. *albicans* cells recovered from the kidneys [17].

Oral administration of 25 mg/kg of tioconazole and miconazole in dogs gave peak concentrations of 4.8 and 0.6 μg/mL, respectively. Mice receiving an oral dose of 25 mg/kg of both agents gave peak serum concentrations of 2.1 μg/mL for tioconazole and 1.4 μg/mL for miconazole [17].

Tioconazole vaginal tablets gave favourable cure rates in patients with vaginal candidiasis [55,56]. Similar results were obtained after a single application with tioconazole 6% ointment. Tioconazole is effective in reducing symptoms and inducing mycological cures in patients with vaginal candidiasis. High cure rates were achieved of 85% without relapse following a 3-, 6-, or 14-day administration schedule of tioconazole (a 100 mg pessary or 5.0 g of 2.0% cream) [44]. Single-dose treatment with tioconazole (300 mg pessary or 6.0% ointment) gave cure rates of 96% [44,56]. Small amounts of tioconazole were absorbed with peak levels ranging from 10–35 ng/mL [57].

In a double blind study patients with dermatophytosis were treated with both tioconazole and miconazole resulting in moderate cure rates, but in patients suffering from skin candidiasis and erythrasma more encouraging results for both products were obtained [58]. In another clinical study in patients with dermatophytosis high cure rates (85 to 95%) were obtained after treatment with tioconazole 1% cream administered once or twice daily [44]. In patients with nail infections and non-responders to treatment with ketoconazole or griseofulvin encouraging results were achieved with a special 28% tioconazole formulation [44].

#### 3.1.7. Sulconazole (**4**, Figure 2)

Sulconazole is a broad-spectrum antimycotic with a high *in vitro* activity against dermatophytes like *T*. *mentagrophytes*, *T*. *rubrum*, *M*. *gypseum*, and *E*. *floccosum* with MIC values ranging from 0.78 to 6.25 μg/mL, which are comparable with that of econazole and clotrimazole (Table 7 and 9) [23]. Against yeasts such as *C*. *neoformans*, *C*. *albicans* the compound displayed MIC’s of 3.13 and 6.25 μg/mL respectively (Table 9), [23]. In an extended *in vitro* study the compound was tested against a large panel of 186 pathogenic *Candida* clinical isolates with MICs ranging from 0.12 to 8.0 μg/mL (Table 7) [33]. Moreover the compound demonstrated pronounced activity against *A*. *fumigatus*, *A*. *flavus* and *A*. *niger* and MIC values ranged from 6.25 up to 25 μg/mL (Table 9) [23].

**Table 9:** *In vitro* antifungal activity of sulconazole [23].

Organism	MIC (μg/mL)
*Trichoiphyton mentagrophytes*	1.56
*Trichophyton rubrum*	6.25
*Epidermophyton floccosum*	0.78
*Microsporum canis*	6.25
*Microsporum gypseum*	12.5
*Candida albicans*	6.25
*Cryptococcus neoformans*	3.13
*Aspergillus fumigatus*	6.25
*Aspergillus flavus*	25
*Aspergillus niger*	12.5

The compound further demonstrated an activity against different Gram-positive *Staphylococci aureus* and *Streptococci epidermis* strains with MIC’s ranging from 0.78 up to 6.25 μg/mL [23]. Inoculum size and pH of the medium influenced the MIC values of the different fungi [23].

*In vivo* sulconazole cream formulations in a variety of dosage schedules were as effective as miconazole in a model of experimental trichophytosis [44]. In other experimental guinea pig and mouse vaginal candidosis model both compounds were equivalent [44]. The data obtained in animal models were confirmed in clinical trials.

Patients with tinea versicolor were successfully treated with 1% sulconazole with complete healing of lesions in 89% of patients [44]. Treatment of dermatophytosis in patients with sulconazole gave encouraging results and in the group of cured patients no relapse occurred [44].

#### 3.1.8. Butoconazole (**8**, Scheme 1).

*In vitro* butoconazole was highly active against dermatophytes, exemplified by MIC values ranging from 0.05 to 5 μg/mL against different strains of *T*. *mentagrophytes*, *T*. *rubrum*, *T*. *tosurans*, *T*. *concentricum*, *M*. *gypseum*, *M*. *canis* and *E*. *floccosum*.

Against yeasts like *C*. *albicans* (ATCC 10231 and ATCC 14053) in broth dilution assay the MIC values were 30 and 10 μg/mL, respectively. The MIC value against *C*. *neoformans* was lower than 1 μg/mL. In another *in vitro* study the compound was tested against a large panel of 186 pathogenic *Candida* clinical isolates with MIC’s ranging from 0.12 to 8.0 μg/mL (Table 9) [33].

Furthermore the compound was active against Gram-positive bacteria with MIC values of 6.25–12.5 μg/mL against *S*. *aureus* and even more active against *S*. *faecalis* and *S*. *pyogenis* with MIC values of 3.12 and 0.0016 μg/mL, respectively. In general, the *in vitro* activity of butoconazole against dermatophytes, yeasts and Gram-positive bacteria was comparable with that of miconazole [7].

No significant differences in activity were seen between butaconazole and both stereoisomers against *C*. *albicans* [26]. The *in vivo* activity of butoconazole was evaluated in an experimentally induced *C*. *albicans* vaginal infection in mice and 80% of the infected animals remained free of infection 3 days later [7].

Activity was demonstrated against the representative dermatophyte *T*. *mentagrophytes* in an *in vivo* guinea pig model; however seven days later only 40% of the animals remained clear [7]. Probably the treatment period was too short to cure the animals. In different clinical studies butoconazole nitrate cream (1 or 2%) proved to be very effective in the treatment of women with vaginal candidiasis. Thirty days after completion of the therapy about 80% of patients were cured [44]. Single dose 2% butaconazole gave significant improvement in women suffering from vaginal candidiasis [59].

#### 3.1.9. Sertaconazole (**2ad**, Figure 2).

Sertaconazole has an *in vitro* broad-spectrum antifungal activity against dermatophytes, yeasts and opportunist filamentous fungi (Table 9). Against dermatophytes like *M. canis*, *T. mentagrophytes*, *T. rubrum* and *M. gypseum* the MIC (μg/mL) ranged from 0.24 to 1.71. For filamentous fungi including *A. fumigatus* and *A. niger* the MIC ranged from 1.41 to 2 μg/mL.

The MIC values ranged from 0.35 to 4.04 μg/mL for *C*. *albicans*, *C*. *pseudotropicalis*, *C*. *parapsilosis*, *C*. *tropicalis*, *C*. *krusei*, *Trichosporum inkin*, *Rodotorula rubra* and *C*. *neoformans* strains [60].

An *in vitro* sertaconazole concentration of 0.06 μg/mL was able to inhibit growth for 90% in 30 different *C*. *albicans* strains. For the 11 strains of *C*. *glabrata*, 18 strains *C*. *parapsilosis* and 15 strains of *C*. *krusei* 0.25 μg/mL was needed to inhibit growth for 90%. *C*. *tropicalis* was less susceptible and a much higher concentration of 2 μg/mL was needed to inhibit growth for 90 % within the tested 20 strains [25].

The compound displays activity against Gram-positive cocci with MIC’s ranging from 0.5 to 16 μg/mL for the different species [61]. In a mouse model of vaginal candidiasis topically applied sertaconazole gave up to 97.7% reduction in cell count of yeasts from vaginal secretions [61].

Sertaconazole gave high concentrations persisting for many hours in vaginal fluids after a single dose of a sertaconazole formulation (cream, tablet or ovule) [25]. Topical treatment with a 2% sertaconazole cream of experimental dermatophytosis in guinea pigs caused by *T*. *mentagrophytes* gave high cure rates [62].

In a limited clinical study patients with dermatophytosis caused by *T*. *rubrum*, *T*. *mentagrophytes*, *M*. *canis*, *E*. *floccosum* and *T*. *schoenleinii* were successfully treated twice daily with 1 and 2% sertaconazole cream [63]*.* The same treatment schedule was effective in patients with *Pityriasis versicolor* infection for both formulations [63]. Patients suffering from different dermatophytoses (tinea cruris, tinea corporis, tinea pedis, tinea manuum, and tinea barbea) responded very well on treatment with sertaconazole cream. High cure rates of 98.3 versus 94.3% respectively were noted after four weeks of treatment in a randomised and comparative study with sertaconazole and miconazole cream. The patients were treated for infections caused by *M*. *canis*, other dermatophytes and *C*. *albicans*. At day 35 a relatively low relapse rate of 4.4% was observed for sertaconazole whereas for miconazole a somewhat higher relapse rate of 11.9% was noted [61].

A single dose treatment with a vaginal tablet of 500 mg gave cure rates of 80% after fourteen days and was comparable with a clotrimazole single dose (500 mg vaginal tablet) [61]. In another study sertaconazole and econazole displayed similar results in patients with vaginal candidiasis, but the percentage of recurrence was lower for sertaconazole [61]. In general sertaconazole is well tolerated [61].

#### 3.1.10. Terconazole (**10**, Scheme 2).

Terconazole is a broad-spectrum antifungal agent (Table 4), displaying activity against different strains of dermatophytes like *M*. *canis*, *T*. *mentagrophytes*, *T*. *rubrum*, *T*. *verrucosum* that were completely inhibited at a concentration of 100 μg/mL after two weeks of exposure in Sabouraud broth. In the same medium different *C*. *albicans*, *C*. *tropicalis*, *T*. *glabrata* and *C*. *neoformans* strains were at least for 90% inhibited at the same concentration. For *A*. *fumigatus* only 2 of the 10 tested strains were completely inhibited, against the other strains no marked inhibition was observed. Testing the compound in Eagle’s Minimum Essential Medium (EMEM) the activity against some *C*. *albicans* strains dramatically increased. In EMEM two *C*. *albicans* strains were virtually complety inhibited at 0.01 μg/mL [64,65] (Table 10). 

**Table 10:** Influence of medium on the antifungal activity of terconazole.

Lowest active concentration of terconazole, μg/mL								
	Sab. broth		Sab.broth+serum		EMEM		EMEM + serum	
	C^(a)^	M ^(b)^	C	M	C	M	C	M
*M*. *canis*	100		100	10	100	10	100	100
*T*. *mentagrophytes*	1		1	0.1	1	0.1	1	0.1
*T*. *rubrum*	100	10	10	1	10	1	10	0.1
*C*. *albicans* str. 1	100	10	100	0.1	0.01		0.01	
*C*. *albicans* str. 2	100		100	0.1	0.1	0.01	10	0.1
*C*. *albicans* str. 3	100		100	0.1	0.1	0.01	10	0.1
C.* tropicalis*	10	1	1	0.1	0.1		1	0.1
*C*. *neoformans*	10	1	1	0.1				
*S*. *schenckii*	100		100	1	100	10	10	
*A*. *fumigatus*	100		100	100	100	100	100	100
*Mucor* species	100		100		10		100	10

^(a) ^Complete inhibition of growth after two weeks of exposure; ^(b)^ Marked inhibition (more than 90% growth inhibition after 14 days of incubation at 25 ºC versus controls.

The most pronounced activity against *C*. *albicans* occurs in EMEM in the presence of serum. The morphogenetic transformation is prevented from the yeast form into the (pseudo)-mycelium form, which is the pathogenic form of this organism in man and in animals, at concentrations of terconazole ranging from 0.008 to 0.04 μg/mL. No detectable inhibition of growth by terconazole was observed up to concentrations of 128 μg/mL against five different strains of lactobacilli, one of the predominant types of bacteria found in the vagina [66].

Topical prophylactic treatment (starting 24h after infection) of vaginal candidiasis in rats infected with *C*.*albicans* gave favourable results at concentrations of 0.5, 0.25 and 0.125% with cure rates of virtually 90, 64, and 78% respectively. In the therapeutic experiment (treatment starting 3 days after infection) the animals were treated for three days twice daily and 79% of the animals were cured or markedly improved [64]. Skin candidiasis in guinea pigs was successfully treated with topical terconazole at concentrations ranging from 0.125 to 0.5%. The majority (96 to 100%) of the animals was cured or markedly improved. 

Treatment with terconazole 0.5% gives 100% cure in *T*. *mentagrophytes* infection in guinea pigs, whereas 1% is necessary to cure *M*. *canis* infections. Orally applied terconazole at 10 mg/kg was effective in 50% of rats with vaginal candidiasis and in guinea pigs with skin candidiasis. A relatively high oral dose of 40 mg/kg was needed to cure or markedly improve a skin infection caused by *M*. *canis* in guinea pigs.

Terconazole as a topically applied agent was found effective in vaginal candidiasis one week after a three-day treatment schedule as an 80 mg suppository. High cure rates of around 90% were observed after a five-day treatment schedule with 0.8% terconazole vaginal cream [67]. Terconazole absorption has been evaluated after intravaginal administration in pregnant and non-pregnant women. The overall outcome in these studies demonstrated that terconazole was minimally absorbed (5–16% of the dose), and was rapidly metabolised [68].

Terconazole cream gave acceptable clinical results in the treatment in patients with vulvovaginal candidiasis caused by non-*C*. *albicans* species with mycological cure rates of 56% [69]. Terconazole is well tolerated as a topical agent and no irritation was observed when a rabbit eye was treated with a cream containing 0.8% of terconazole [70].

### 3.2. Oral treatment of superficial and deep mycoses

#### 3.2.1. Ketoconazole (**9w**, Scheme 2) [71]

Ketoconazole is the first broad-spectrum antifungal agent with oral activity and can be used to treat superficial and deep mycoses. *In vitro* the compound displays activity against a wide range of fungi including dermatophytes, yeasts and other fungi (Table 7) and for a complete overview of its antifungal spectrum see Heel *et al.* [71]). In the presence of 10% inactivated bovine serum the activity remains equal or markedly improves. In case of *C*. *albicans*, *M*. *canis* and *A*. *fumigatus* the activity improves with a factor ten. Ketoconazole is highly active against *T*. *mentagrophytes*, which is completely or markedly inhibited at 0.1 μg/mL. *T*. *rubrum*, *C*. *neoformans*, *C*. *tropicalis*, *S*. *schenkii*, *S*. species and *P*. *verrucosa* all are inhibited at 1 μg/mL. At a concentration of 10 μg/mL *M*. *canis* and *C*. *albicans* are inhibited. In another *in vitro* study the compound was tested against a large panel of 186 pathogenic *Candida* clinical isolates with MIC’s ranging from 0.12 to >64.0 μg/mL (see Table 7) [33].

Additionally the *in vitro* activity of ketoconazole was studied in mixed cultures of leukocytes and *C*. *albicans*. Normally both polymorphonuclear cells and macrophages avidly engulf added *C*. *albicans* yeast cells. However the eradication was not successful even when only 450 yeast cells were added to 3x10^6^ leukocytes. The major route for the fungus to escape in the presence of leukocytes to intracellular killing seems to be the switch into the mycelial form. In the presence of ketoconazole the outgrowth of and formation of mycelia in culture was prevented at very low concentrations (0.01 μg/mL). Addition of ketoconazole in concentrations ranging from 0.01–10 μg/mL gave the leukocytes the opportunity to eradicate the yeasts completely. At much higher concentrations of 100 μg/mL ketoconazole gave some toxic effects [72]. Against freshly isolated strains of *M*. *furfur* isolated from pityriasis versicolor lesions ketoconazole was highly active at concentrations of 0.8 μg/mL [73].

In the prophylactic oral treatment (starting on the day of infection) of vaginal candidiasis in the rat, a daily dose of 1.25 mg/kg only had a marginal effect in contrast with 2.5 mg/kg being highly effective, whereas 5 mg/kg gave total protection [9]. In the curative treatment (therapy starting three days after infection), nearly all animals were cured at daily dose levels of 5 mg/kg for five consecutive days. Lower doses were ineffective even on prolonged treatment up to 11 days.

Against induced cutaneous candidiasis in guinea pigs caused by *C*. *albicans* a pronounced activity was observed at a dose of 2.5 mg/kg, and an almost complete protection was achieved at 10 mg/kg [9,28]. Furthermore ketoconazole had a pronounced effect on induced dermatophytosis with *M*. *canis* and *T*. *mentagrophytes* as causative agents in guinea pigs [28]. In experimental murine coccidioidomycosis it appeared that a dose of 40 mg/kg daily for 2.5 weeks was completely protective in mice infected with a number of *C*. *immitis* cells that normally kills more than 60% of untreated animals. With higher doses and prolonged therapy, eradication of all pathogens from the host could be achieved [74,75].

Disseminated cryptococcosis and histoplasmosis in athymic nude mice gave life span prolongation after a two- to five-week course with ketoconazole in a dose-dependent fashion [76]. In human volunteers the absorption of ketoconazole (200 mg) was rapid after oral administration of a tablet, suspension or solution with mean maximum concentrations of the drug in plasma of 4.2, 5.0, and 6.2 μg/mL attained at 1.7, 1.2 and 1.0 h post-administration [77]. After absorption ketoconazole undergoes extensive hepatic metabolism [78].

Patients suffering from paracoccidioidomycosis (South American Blastomycosis) were successfully treated with ketoconazole and remained in remission for 12–24 month after therapy [79]. Chronic mucocutaneus candidiasis (CMC) is a heterogeneous group of cellular immunodeficiency states that result in chronic infection by *C*. *albicans* of the skin and nails as well as oral, esophageal and genital mucosa. Many patients with this affection responded well on ketoconazole therapy resulting in a marked improvement or in some cases even in a cure. Because of an underlying immunodeficiency state many patients needed continued maintenance therapy [79,80].

Oral thrush caused by *C*. *albicans* responded very well to ketoconazole therapy and was associated with high cure rates [79]. Ketoconazole has been shown to be very effective against dermatophyte infections caused by *T*. *rubrum* that were resistant to other therapies including griseofulvin [81]. Vulvovaginal candidiasis could be treated very well with oral ketoconazole with cure rates including marked improvements of 81% [79]. Treatment of chromomycosis with ketoconazole resulted in either moderate to markedly improvement or clinical cure in 77% of patients [79]. In candiduria similar results were observed. Infections caused by *C*. *albicans* gave better response rates than those caused by *T. glabrata* [79].

Ketoconazole also was an effective treatment of patients with disseminated or progressive histoplasmosis, different forms of systemic and disseminated candidiasis, chromomycosis, sporotrichosis, North American Blastomycosis and coccidiodomycosis [79,82]. Oral ketoconazole gave high cure rates of more than 90% in the treatment of pityriasis versicolor [79].

In general ketoconazole is well tolerated. In some patients treated for fungal infections of the nail, however, symptomatic hepatic reactions during ketoconazole therapy have been reported, but in general symptoms usually subsided when treatment was discontinued [81]. 

Ketoconazole is a potent inhibitor of CYP3A4. Following oral administration of ketoconazole the following adverse effects have been reported including nausea, vomiting (in approximately 3% of patients), abdominal pain (1.2%), and pruritis (1.5%). Less than 1%, of patients has experienced headache, dizziness, fever, chills, diarrhea, gynecomastia, impotence, thrombocytopenia, leukopenia, and hemolytic anemia. Most of these effects were transient and discontinuation of therapy was not required. Some serious adverse effects such as anaphylaxis, hypersensitivity reactions and hepatic toxicity have been reported in rare cases [173].

#### 3.2.2. Itraconazole (**11c**, Scheme 2)

Under standard conditions in Sabouraud medium itraconazole had a moderate activity against the dermatophytes *T*. *rubrum* and *T*. *mentagrophytes*, which were totally inhibited at concentrations of 10 μg/mL [83]. When DMSO was added to the medium at pH 7.4 the activity of the compound was markedly improved against dermatophytes, *Candida* species, *C*. *neoformans*, *A*. *fumigatus*, *S*. *schenckii*, and *Ph*. *verrucosa* [83].

Additionally itraconazole *in vitro* activity was confirmed in Kimmig agar against 148 isolates of pathogenic fungi, in particular against *C*. *neoformans* [85]. *C*. *neoformans* infections were rare prior to the onset of the AIDS epidemic, but have now become a leading cause of morbidity and mortality in AIDS and other immunocompromised patients [84]. The prevalence of cryptococcal infections has been reported to be 5–10% among AIDS patients in the United States and is even higher in Africa [84]. Against other yeasts like *C*. *albicans*, *C. parapsilosis*, *C*. *tropicalis*, *Candida* species and *C*. *glabrata* the MIC’s varied widely ranging from 0.063 to >128 μg/mL. For a complete overview of the antifungal spectrum of itraconazole and comparison with other azoles such as fluconazole, posaconazole, voriconazole and amphotericn B (see Aperis *et al.* [107]).

The dimorphic fungi like *B*. *dermatitidis* and *H*. *capsulatum* were very sensitive with MIC values ranging between 0.063 and 0.13 μg/mL, whereas *S*. *schenckii* was less sensitive with an MIC of 1–4 μg/mL for all strains. Against dermatophytes including *E*. *floccosum*, *M*. *canis* and *Trichophyton* species the MIC ranged from 0.063 to 64 μg/mL. Against dematiaceae the MIC ranged from 0.063 to >128 μg/mL. *A*. *fumigatus* and *A*. *flavus* were very sensitive to itraconazole with MIC’s varying from 0.063 to 2 μg/mL, whereas *P*. *boydii* was clearly less sensitive with an MIC from 16 to 32 μg/mL for all strains. Zygomycetes were inhibited at concentrations ranging from 0.063 to >128 μg/mL [85]. In a variety of fungi itraconazole induced irreversible damage, depending on the species tested, the time of incubation and morphogenetic form of the fungus tested. Itraconazole achieved irreversible damage against the following fungi including *C*. *albicans*, *C*. *neoformans*, *P*. *ovale*, *T*. *rubrum*, *P*. *brasiliensis* and *A*. *fumigatus* [86].

In vaginal candidiasis (therapy starting 3 days post-infection) in the rat itraconazole at a daily dose of 10 mg/kg cured all animals, but at a daily dose of 2.5, 1.25, and 0.63 mg/kg partial cure rates were achieved [87]. Treatment of experimental microsporosis (treatment starting the day of infection) caused by *M*. *canis* in the guinea pig gave encouraging results with a cure rate of 12/15 animals at a daily dose of 1.25 mg/kg [11]. Topically applied itraconazole was effective at concentrations of 0.125% in the same infection model.

In systemic candidiasis in guinea pigs caused by *C*. *albicans* an oral dose of 5 mg/kg was needed to cure or markedly improve all animals with virtually negative cultures isolated from the skin and kidneys. Guinea pigs infected with *S*. *schenckii* or with *H*. *duboisii* responded well to treatment with 20 and 10 mg/kg of itraconazole, respectively.

In mice with aspergillosis the survival time was doubled after treatment with 40 mg/kg and was even tripled with 80 mg/kg compared to untreated controls. Following oral treatment with itraconazole high cure rates were observed in intracerebral cryptococcosis in mice resulting in a similar outcome when treatment started simultaneously or when it started 48 h after induction of the infection. The anticryptococcal activity of itraconazole was also very pronounced in guinea pigs [87].

Pharmacokinetic studies in volunteers were performed on days one and 15 at the following dosages, 100 mg once daily, 200 mg once daily, and 200 mg twice daily. On each study day, itraconazole was administered with a standard meal. Peak plasma levels of 110 ng/mL at 2.8 h (100 mg once daily), 272 ng/mL at 3.0 h (200 mg once daily), and 553 ng/mL at 3.4 h (200 mg twice daily) were achieved. At day 15 the following peak serum levels of 412 ng/mL at 3.0 h (100 mgonce daily), 1,070 ng/mL at 4.4 h (200 mg once daily), and 1,980 ng/mL were measured, respectively. The elimination half-lives on days 1 and 15 were 15 and 34 h (100 mg once daily), 20.7 and 36.5 h (200 mg once daily), and 25 and 41.7 h (200 mg twice daily), respectively [174].

Itraconazole absorption is enhanced by the intake of food [88]. The oral solution delivers higher concentrations in the serum than the capsules [89]. Itraconazole is a substrate for and an inhibitor of CYP3A4 which is responsible to metabolize itraconazole [173].

The clinical pharmacokinetic and metabolic behaviour of itraconazole is rather complex, because it undergoes an *in vivo* biotransformation into a major metabolite hydroxy-itraconazole (hydroxylation in the 2-position of the *s*-butyl group), which exerts a similar antifungal activity [88]. Itraconazole (with a *cis*-configuration in the dioxolane ring) has three chiral centers and is a mixture consisting of four stereoisomers. From *in vitro* experiments with heterologously expressed CYP3A it became clear that only (2*R*,4*S*,2′*S*)- and (2*R*,4*S*,2′*R*)-itraconazole were metabolised by CYP3A4 to hydroxy-, keto-, and *N*-desalkylitraconazole, respectively. On the other hand when (2*S*,4*R*,2′*R*)- or (2*S*,4*R*,2′*S*)-itraconazole was incubated with CYP3A4, there was no metabolization or loss of substrate. Notwithstanding all these differences in metabolism patterns, all four stereoisomers induced a type II binding spectrum with CYP3A4. *In vivo* pharmacokinetic studies in healthy volunteers treated for several days with 10 mL of 10 mg/mL oral solution of itraconazole revealed that at day 1 the stereoisomers (2*R*,4*S*,2′*S*)- and (2*R*,4*S*,2′*R*)-itraconazole, which were metabolised by CYP3A4, had a higher oral clearance and smaller AUC’s than the other stereoisomers (2*S*,4*R*,2′*R*)- and (2*S*,4*R*,2′*S*)-itraconazole. At day one the peak level in one volunteer was 99 nmol/L for the (2*R*,4*S*)-ITZ and 302 nmol/L for the (2*S*,4*R*)-ITZ stereoisomer pair, respectively. At day 7 after multiple dosing of itraconazole the peak concentrations were more similar with values of 302 nmol/L for (2*R*,4*S*)-ITZ and 302 nmol/L for (2*S*,4*R*)-ITZ stereoisomer pair, indicating that stereoselectivity was diminished, probably due to autoinhibition of CYP3A4 [90]. Itraconazole is primary metabolised in the intestine to its principle metabolite hydroxy itraconazole, which is subsequently metabolised to the keto derivative and finally to the *N*-desalkylitraconazole [90].

Historically, treatment of cutaneous fungal infections was based on topical therapy, which still occupies a prominent place in the treatment of acute lesions of limited extent. However, these topical agents notwithstanding their intrinsic *in vitro* potency, are inconvenient for use in extensive infections and in these cases compliance is a real issue. Therefore systemic antifungal agents offer a therapeutic alternative and convenience in chronic infections and in cases resistant to topical treatment or with an extensive involvement of the skin infection [91].

Itraconazole is a highly lipophilic drug with a high affinity for keratinous tissues, in which the concentration is many fold higher than in the serum. In tinea corporis and tinea cruris good to excellent results were achieved with 200 mg of itraconazole once daily. Short treatment schedules of 200 mg twice daily were very successful in tinea pedis patients resulting in mycological cure rates of 85% and a corresponding clinical response at four weeks after treatment.

*In vitro* itraconazole has a high activity against *M*. *canis* and other dermatophytes involved in scalp ringworm infections. Children with tinea capitis were treated with 5 mg/kg/day for six weeks and two months after the end of treatment, 89% of the children were cured [91]. The compound gives high cure rates of up to 95% in the treatment of tinea versicolor caused by *M*. *furfur* [44]. Itraconazole in general is well tolerated with side effects reported in about 7% of patients treated for dermatologic indications. The adverse effects are mostly minor and primary consist of gastric upset and headache [91].

One of the most promising approaches to the management of onychomycosis is based on the concept of intermittent or pulse therapy with itraconazole [92]. In clinical trials with itraconazole in vaginal candidiasis and in pityriasis versicolor there were indications that 2 to 3 days treatment in the first infection and 5 days treatment in the latter gave high cure rates [93].

In immunosuppressed patients (organ transplantation and HIV-infected people) severe mucosal candidiasis can be treated very well with itraconazole with a response rate of 90%, and similar response rates are obtained with fluconazole [94]. In *Candida* esophagitis fluconazole and itraconazole both are indicated [94]. Relapsed vulvovaginal candidiass in patients with an underlying disease like diabetes mellitus can be treated with fluconazole 150 mg pro week or with itraconazole 100 mg daily [94].

Patients with pulmonary or disseminated blastomycosis and histoplasmosis gave cure rates of 95% after treatment with itraconazole (200–400 mg daily) [95]. HIV-infected patients with histoplasmosis not being acutely ill and not having meningeal involvement can be treated with itraconazole.

The drug can also be used in the treatment of lymphocutaneus or cutaneous sporotrichosis and in pulmonary sporotrichosis, paracoccidiodomycosis and non-invasive aspergillosis [44,95]. In more complicated affections the compound was less successful, for example in the treatment of invasive aspergillosis (62%), meningeal cryptococcosis (50%) and aspergilloma (44%) [44].

Furthermore, itraconazole is effective in coccidioidomycosis [96]. Improvement in chromomycosis caused by *Cladosporum carrioni* and mycetoma caused by *Madurella grisea* was obtained in itraconazole treatment [44]. Encouraging results were observed in candidiasis (systemic and mucocuteneus) [44]. Itraconazole was found to be active in the prevention of fungal infections in patients with severe granulocytopenia. In the prevention of *Aspergillus* infections itraconazole was particularly active [44].

In general itraconazole is well tolerated in patients. The most reported adverse effects include gastrointestinal disorders such as dyspepsia, abdominal pain, nausea, and diarrhea. Occasionally gastritis, hepatitis and some rare cases of liver failure have been reported. Furthermore monitoring of liver enzymes is recommended after continuous administratin of itraconazole for over one month [175].

#### 3.2.3. Fluconazole (**15a**, Scheme 4)

With fluconazole in particular *in vitro* antifungal data are not predictive for *in vivo* activity. For a complete overview of the antifungal spectrum of fluconazole and comparison with other azoles such as itraconazole, posaconazole, voriconazole and amphotericn B see Aperis *et al.* [107].

It was noted that at physiologic pH fluconazole was significantly less active *in vitro* than ketoconazole against 35 isolates of *C*. *albicans* [44]. In another assay it was demonstrated that fluconazole was less active than ketoconazole and amphotericin B against yeasts and other filamentous fungi when tested in both liquid and solid medium. In an agar dilution assay fluconazole was more active at pH 7 to 7.5 than at lower pH [44]. Fluconazole is active against *Candida* species (Table 7 and 12) and *C*. *neoformans*, is less active against dermatophytes and displays no activity against *Aspergillus* species [44]. Fluconazole was fungistatic and not fungicidal when tested either against *Candida* species in the stationary or in the logarithmic phase [97]. Fluconazole was evaluated for *in vitro* activity by using four different tests [98].

Clotrimazole and ketoconazole both reduced proliferative responses of lymphocyte suspensions to mitogens but fluconazole showed no anti-lymphocyte effect [99]. Fluconazole resistant *C*. *albicans* [100] and *C*. *neoformans* [101] showed low level resistance to itraconazole and voriconazole.

The relatively poor *in vitro* antifungal activity contrasted with the high *in vivo* activity in experimental fungal infection models in animals and is mainly based on mouse models. Fluconazole was effective in the treatment of experimental cryptococcal meningitis in mice, following intranasal and intravenous administration of *C*. *neoformans*. Moreover fluconazole was able to control cryptococcal meningitis after intracerebrally administered cells of *C*. *neoformans* in the same model [44].

Oral treatment with high-dose of fluconazole prolonged survival and reduced the number of colony-forming units of fungus in the brain of mice that had been challenged intracerebrally with endospores of *C*. *immitis* [44]. Fluconazole is effective in the treatment of experimental histoplasmosis caused by *H*. *capsulatum* in mice. Subcutaneously administered fluconazole at a daily dose of 50 mg/kg during 21 days gave 100% survival in mice with experimental blastomycosis [44]. Notwithstanding the overall survival fluconazole was not able to clear the lungs from fungus.

In normal mice with systemic infection of *C*. *albicans* fluconazole administered for 30 days was able to prolong life to more than 90 days and could be associated with a clearance of *C*.*albicans* in the kidneys of up to 60% of the animals [44]. Immunosuppressed mice with intestinal candidiasis delivered culture negativity in faeces after treatment with fluconazole for 3 days [44]. Treatment with fluconazole resulted in prolonged survival times in rats with systemic candidiasis.

Fluconazole was effective in the treatment of vaginal candidiasis in mice [44]. Contradictory results have been published in the treatment of aspergillosis in mice with fluconazole [44]. Fluconazole is an inhibitor of CYP3A4, CYP2C9, and CYP3C19, respectively [173].

The bioavailability of fluconazole is high after oral administration (1 mg/kg) in mice, rats, dogs and humans [44]. Elimination half-lives of 22 h were observed in humans. Oral administration of fluconazole gives excellent cerebrospinal fluid concentrations. The major route of elimination of fluconazole is renal clearance with 70% excretion of unmetabolised drug in the urine.

A single dose treatment of vaginal candidiasis with fluconazole (150 mg) gave a mycological cure rate of over 80% after one week [44]. Patients with oropharyngeal candidiasis of which the majority of them was positive for the human immunodeficiency virus could be successfully treated [44].

Cryptococcal meningitis can be treated with fluconazole as a maintenance therapy after an induction therapy with amphotericin B and flucytosin, and in AIDS patients continuing prophylactic therapy with fluconazole relapse rate reduced to 5% [94].

Fluconazole treatment gave promising results in patient with coccidioidal meningitis, a disease with a high morbidity and mortality [102]. In general fluconazole is well tolerated and the most common adverse effects of fluconazole include headache, nausea, abdominal pain, diarrhea, dyspepsia, dizziness, and taste perversion. Furthermore some cardiotoxic effects have been reported [175].

#### 3.2.4. Voriconazole (**16f**, Scheme 4)

Voriconazole is a triazole butanol-2 derivative and is a broad-spectrum antifungal agent. For a complete overview of the antifungal spectrum of voriconazole and comparison with other azoles such as fluconazole, itraconazole, posaconazole, and amphotericn B see Aperis *et al.* [107].

*In vitro* voriconazole is very potent against *C*. *albicans* (660 isolates, MIC_90_, 0.06 μg/mL), *C*. *parapsilosis* (221 isolates, MIC_90_, 0.12 μg/mL), and *C*. *tropicalis* (139 isolates, MIC_90_, 0.25 μg/mL). Isolates of *C*. *krusei* (33 isolates, MIC_90_; 1.0 μg/mL), and *C*. *glabrata* (217 isolates, MIC_90_, 2.0 μg/mL) were somewhat less susceptible to voriconazole [84]. The *in vitro* activity against *C*. *neoformans* (64 isolates) has been confirmed by different groups and the MIC ranges from 0.0313 to 0.5 μg/mL with an MIC_90_ value of 0.25 μg/mL. Voriconazole is also active against other fungal species like *B*. *dematitidis* (MIC range 0.03–1 μg/mL), *H*. *capsulatum* (MIC range 0.03–1 μg/mL), *C*. *immitis* (MIC range 0.03–0.125 μg/mL), and *Pseudoallescheria boydii* (MIC_50_ 0.12 μg/mL) [84].

Against *Fusarium* and *Scedosporium* species the *in vitro* activity of voriconazole is at least similar or superior to that of itraconazole and posaconazole, respectively [84,107]. Furthermore voriconazole displays a high *in vitro* activity against *Aspergillus* species with an MIC_90_ ranging from 0.125 to 2 μg/mL [84]. Against voriconazole-resistant laboratory *Aspergillus fumigatus* strains a 16.9 fold increase of MIC was noted, whereas for itraconazole and posaconazole an MIC increase of 2.76 and 1.42 was observed, respectively, suggesting that a low level of cross-resistance exist between both classes of azole antifungals [104]. Similar amounts of voriconazole were accumulated in the susceptible and resistant isolates. Consistent with its *in vitro* potency; voriconazole is highly effective against various fungal infections in guinea pigs. The guinea pig was chosen because in this species the drug achieves systemic exposures that are comparable to those observed in man [105].

Voriconazole is well absorbed with maximum concentrations being reached in 2 h after oral administration in normal human subjects [106]. Voriconazole has a mean serum half-life of about 6 h, and steady-state concentrations in the serum are reached within 5 to 7 days at dosages of 200 mg twice daily [106].

Voriconazole is metabolised in the liver primarily by CYP2C9, to a much lesser extend by CYP2C9, and CYP3A4 by N-oxidation. Its metabolites have little clinical effect, excretion does not depend on renal function [89,107] and the metabolites are mainly excreted via the bile and urine [106]. Voriconazole is an inhibitor of CYP2C19, CYP2C9 and CYP3A4. Voriconazole is commercially available in both oral and intravenous form. The bioavailability after oral administration of voriconazole is > 90% on an empty stomach and is not affected by changes in gastric pH, but on the other hand it is negatively affected by the presence of food [107].

Therapeutic levels of voriconazole can be achieved in the non-inflamed human eye [107]. Immunosuppressed patients seropositive for HIV suffering from esophagitis due to *Candida* species were treated twice daily with 200 mg of voriconazole to give cure rates of 98.3%. In the same study fluconazole 200 mg once daily gave a cure rate of 95.1%. In addition voriconazole was effective in patients with fluconazole-resistant esophageal candidiasis [107,108].

Treatment with voriconazole (mean dose 400 mg daily) for 50 (mean) days gave a favourable evolution in patients with invasive candidiasis refractory to former treatments [103]. Voriconazole versus amphotericin B followed by fluconazole was evaluated for candidaemia in non-neutropenic patients (370). Both treatment regimens had a similar efficacy against all *Candida* species except *C*. *tropicalis* in which voriconazole was the superior treatment. Similar mortality rates of 36% and 42% were observed with voriconazole versus amphotericin B-fluconazole treatment, respectively [107].

In some of 25 non-neutropenic patients with chronic invasive aspergillosis (of whom approximately 50% treatment failures with itraconazole or amphotericin B) a favourable response was observed after treatment with voriconazole. A number of patients experienced mild or moderate visual disturbances (enhanced perception of light) and monitoring levels of voriconazole is recommended [106,107].

Therapeutic drug monitoring of voriconazole can help to predict treatment outcomes, visual disturbances or transaminase abnormalities. Visual disturbances or clinical significant transaminase abnormalities have occurred in 30 and 12.7% of patients, respectively. It is estimated that for every 1μg/mL increase in serum level of voriconazole up to 9 μg/mL, the odds of experiencing visual disturbances increase by 4.7% [89].

Invasive aspergillosis in immuno-compromised patients is life-threatening. Treatment with voriconazole of patients with invasive aspergillosis achieved clinical remission of 52% and survival in 72% of patients [94,107,108]. A study in 86 cases of cerebral aspergillosis, in majority refractory to previous treatments revealed a success rate in 34% of cases after treatment with voriconazole [108].

In patients with *Scedosporium* or *Fusarium* infection refractory or intolerant to previous treatment encouraging results were achieved [108]. Cryptococcal meningitis recalcitrant to previous treatments responded for 39% after treatment with voriconazole [108]. Visual disturbances, fever nausea, vomiting, diarrhea, headache, abdominal pain, and respiratory disorders are the most common adverse effect of voriconazole. Skin eruptions in approximately 7% of patients were observed during clinical trials [173].

#### 3.2.5. Posaconazole (**12l**, Scheme 3)

Posaconazole is a pure enantiomer and is structurally very close to itraconazole with both molecules containing a triazole and a triazolone ring. The compound is a broad-spectrum antifungal agent active against dermatophytes, yeasts and filamentous fungi. For a complete overview of the antifungal spectrum of posaconazole and comparison with other azoles such as fluconazole, itraconazole, voriconazole, and amphotericn B see Aperis G. *et al* [107].

Posaconazole was tested against 30 different clinical isolates of dermatophytes including 16 strains of *M*. *canis*, six strains of *T*. *rubrum*, four strains of *T*. *mentagrophytes*, two strains of *E*. *floccosum*, and one strain of *T*. *verrucosum* and *M*. *gypseum*, respectively. The MIC_90_ ranged from 0.06 to>4.0 μg/mL [109].

Furthermore posaconazole was highly active against bloodstream isolates of *Candida* species (1300 strains) with an MIC_90_ of 1.0 μg/mL. The most susceptible fungus among the *Candida* species was *C*. *albicans* (660 strains) with an MIC_90_ of 0.06 μg/mL and the least sensitive fungus was *C*. *glabrata* (217 strains) with an MIC_90 _of 4 μg/mL. Posaconazole demonstrated high *in vitro* activity against *C*. *parapsilosis*, *C*. *tropicalis* and *C*. *krusei* with an MIC_90_ ranging from 0.12 to 0.5 μg/mL [84]. Against *C*. *dubliniensis*, a newly recognized fungal pathogen causing mucosal disease in AIDS patients posaconazole displayed an MIC_90_ value of less than 0.5 μg/mL. Higher MIC values for posaconazole were noted for *C*. *dubliniensis* isolates having a lower susceptibility against fluconazole. The MIC_90_ value for posaconazole against *Aspergillus* species ranges from 0.12 to 0.25 μg/mL with *A*. *fumigatus* being the most susceptible species [110]. Moreover the compound was very potent against *P*. *boydii* (MIC_50_, 1 μg/mL) and against *Rhizopus* spp. (MIC_50_, 1 μg/mL) [110]. Against different *C*. *neoformans* strains the MIC values ranged from 0.063 to 0.125 μg/mL. Moreover posaconazole is very potent against *B*. *dermatitidis*, *H*. *capsulatum* and *C*. *immitis* with MIC’s ranging from 0.0047 to 4 μg/mL [84,107,111,112].

The compound is fungicidal against *C*. *inconspicua*, *C*. *kefyr*, *C*. *krusei*, *C*. *lusitaniae*, and *C*. *neoformans*, and generally against *Aspergillus* species. Fungistatic activity has been observed against *C*. *albicans*, *C*. *guilliermondii*, *C*. *glabrata*, *C*. *parapsilosis*, and *C*. *tropicalis* [1].

Posacoconazole gave positive test results in murine models of intratracheal histoplasmosis in immunocompetent and in immunocompromised mice [113,114]. Life span increased in mice infected with *B*. *dermatitidis* after treatment with posaconazole (25, 5, or 1 mg/kg/day of body weight). Sterilisation of the lungs only occurred in the high-dose group [111]. Effective cures in mice infected with *C*. *immitis* could be achieved after treatment with posaconazole at a daily dose of 10 and 25 mg/kg, respectively [112].

Moreover posaconazole showed excellent activity against *A*. *fumigatus* and *A*. *flavus* in a pulmonary mouse infection model [115]. The compound was also very efficacious against fluconazole-susceptible, -susceptible dose-dependent or -resistant *C*. *albicans* strains in immunocompetent or immunocompromised mouse models of systemic infection [115]. Posaconazole was further evaluated in a temporarily neutropenic mouse model of invasive aspergillosis with an itraconazole-susceptible and an itraconazole-resistant isolate of *A*. *fumigatus*. In both groups of mice posaconazole at daily doses of 5, 10 and 25 mg/kg, respectively, gave prolongation of survival compared to controls. In the highest dose (25 mg/kg) group all animals infected with the itraconazole-resistant *Aspergillus* strain survived. In the mice infected with the itraconazole-sensitive *Aspergillus* virtually all the animals survived treated with the 3 different doses [116]. Posaconazole prolonged survival and reduced lung tissue counts of *A*. *fumigatus* in mice immunosuppressed with corticosteroids [117].

High doses of 50 and 100 mg/kg daily were successful in the treatment of immunocompetent mice infected with *Fusarium solani*, more and more causing a life-threatening disease in patients with compromised immune responses [118].

The pharmacokinetics of posaconazole was evaluated in studies involving mice, rats, rabbits, dogs, and cynomolgus monkeys via intravenous or oral administration as a solution in hydroxypropyl-β-cyclodextrin or via oral administration as a suspension in 0.4% methylcellulose. In all species posaconazole was bioavailable. Oral bioavailability was generally higher with the cyclodextrin (range, 52–100%) solution than with the methylcellulose suspension (range, 14–48%). Bioavailability was the highest in mice (100%), followed by dogs (72%), rats (66%) and monkeys (52%). Increasing oral doses (mice, rats, and dogs) gave higher serum concentrations and was dose-related. Food intake resulted in a fourfold increase in serum concentration of posaconazole in dogs [119].

In healthy volunteers the bioavailability of posaconazole increases with co-administration of a liquid nutritional supplement containing 14 g fat [120]. Absorption is not affected by gastric pH but in contrast to voriconazole, ingestion of a high-fat meal leads to a 400% increase in the relative oral bioavailability of posaconazole [107]. A dose escalation study learned that half-life of posaconazole increased after increasing the dose. Posaconazole undergoes significant enteropatic recirculation with the primary route of elimination via the bile and the faeces [84]. Oral posaconazole has a large volume of distribution and a long terminal-phase half-life of 25–31 h. Like with itraconazole, plasma protein binding is high. Metabolization of posaconazole takes place in the liver, where it undergoes glycoronidation and is converted to biologically inactive metabolites. A small amount of the parent compound together with multiple glycoronidated metabolites is excreted in the urine up to 14% of the dosage, and 77% of the parent compound is excreted in the faeces [107]. Posaconazole is not metabolized by CYP enzymes [1]. Posaconazole is a substrate and potent inhibitor of CYP3A4 [173]

Plasma levels of 4 μg/mL are achieved after oral administration of posaconazole at 400 mg twice daily for 14 days [107]. A sensitive and selective high-performance liquid chromatography (HPLC) method has been developed based on ultra-violet detection for the simultaneous determination of voriconazole and posaconazole [121].

In a double-blind study no significant differences were found in patients receiving immunosuppressive therapy for Graft-Versus-Host Disease after allogenic bone marrow transplantation with main underlying conditions chronic and acute myelogenous leukemias between fluconazole and posaconazole to prevent invasive fungal infections [1]. In a large double-blind study with participation of 437 HIV-infected patients with esophagial candidiasis posaconazole and fluconazole gave success rates of 77–87% and 89%, respectively. Mycological eradication was achieved in 36–40% of the patients treated with posaconazole versus 50% with fluconazole [107].

In a randomized clinical trial the activity of posaconazole was compared with fluconazole in the treatment of HIV-associated oropharyngeal candidiasis in 339 patients. Both agents gave clinical cure rates of 82–83% and a mycological cure rate of 62% [107].

The efficacy of posaconazole was studied in a group of 39 patients with proven or probable CNS infections, among them were 29 with cryptococcal meningitis. All of them had invasive infection refractory or intolerant to conventional therapies, such as flucytosine, fluconazole, itraconazole or amphotericin B. A positive outcome in 14 patients with cryptococcal meningitis was achieved after treatment with posaconazole [107]. Posaconazole gave impressive results in the treatment of recalcitrant zygomycosis with response rates of 60–100% [107].

In therapy failures of disseminated pulmonary histoplasmosis, previously treated with amphotericin B, itraconazole, liposomal amphotericin B, fluconazole or voriconazole posaconazole treatment gave a positive outcome in six of the seven patients [107].

Posaconazole oral suspension (800 mg daily in divided doses) was investigated as monotherapy in an open-label, multicenter study in patients (238) with invasive aspergillosis and other mycoses who were intolerant or refractory to conventional therapy (80% of patients), mostly amphotericin B. Response rates were: 42% (aspergillosis), 39% (fusariosis), 69% (coccidioidomycosis), 48% (candidiasis), 48% (cryptococcosis), 81% (chromoblastomycosis or mycetoma) and 56% (zygomycosis) [122,123,124].

Treatment with posaconazole of paediatric patients with various invasive fungal infections like aspergillosis, candidiasis, coccidioidomycosis, fusariosis, and zygomycosis, refractory or intolerant to amphotericin B or itraconazole resulted in a successful outcome of 45% in patients [107].

Antifungal therapy in critically ill patients often requires long treatment periods. In general prolonged treatment with posaconazole was associated with a favourable safety profile in patients with refractory invasive fungal infections, being seriously ill [124].

In general posaconazole is well-tolerated, the most common side-effect are gastrointestinal in nature, including nausea (7–8%), and diarrhea (3–11%) which rarely led to discontinuation of therapy. Some other common adverse effects were vomiting (4–7%), headache (2–8%), and liver enzyme elevations (2–3%) [1].

## 4. Mode of Action

### 4.1. Ergosterol pathway inhibition

**Scheme 5:**
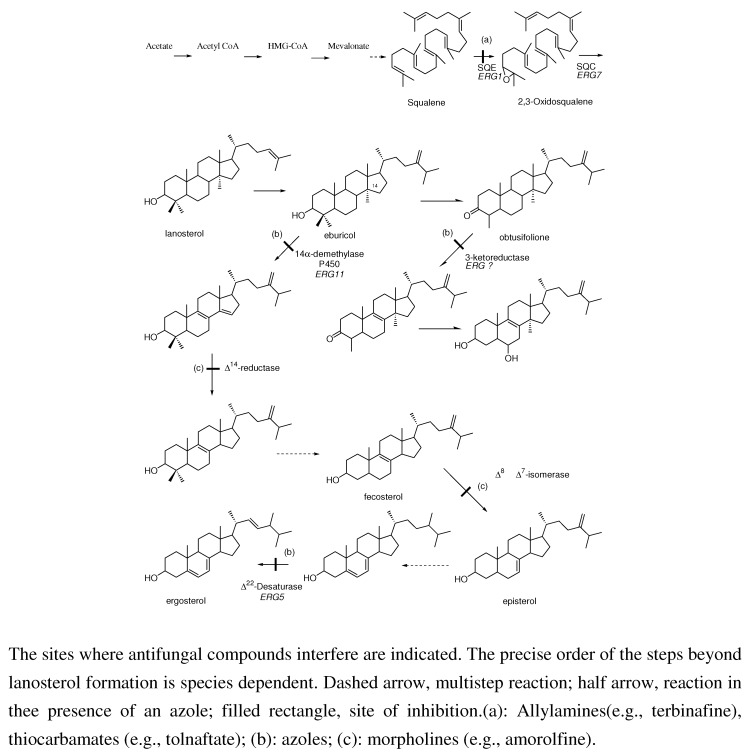
Synthesis of ergosterol.

The antifungal activity of azoles arises from a complex multi-mechanistic process initiated by the inhibition of the cytochrome P450s catalyzing the 14-α-demethylation step, encoded by ERG11 (CYP51), and the Δ^22^-desaturase, encoded by ERG5 (CYP61) [162,176]. The demethylase protein CYP51 is the product derived from the ERG11 gene (in *C*. *albicans*) and is present in virtually all yeasts and moulds with exception of *Pneumocystis* and *Pythium* species [125]. The CYP51 enzyme belongs to the family of cytochrome P-450 enzymes, and is involved in the biosynthesis of ergosterol (Scheme 5), which is a major constituent of the cell membrane of fungi [125]. In mammalian cells CYP51 plays a similar role in the synthesis of cholesterol. The antifungal activity of azoles is based on binding to a cytochrome P-450 (CYP51), which plays an important role in the 14α-demethylation of lanosterol (in *Saccharoromyces cerevisae*, *C*. *glabrata*, and mammalian cells) or of eburicol (in filamentous fungi, the yeast form of *H*. *capsulatum*, *C*. *neoformans*, and a number of *C*. *albicans* strains). Cytochrome CYP51 enzyme is membrane bound (to the mitochondrial inner membrane and the membrane of the endoplasmatic reticulum) in eukaryotes, whereas in prokaryotes the protein is present in soluble form [126].

Azole antifungal agents are inhibiting the oxidative conversion of lanosterol to ergosterol which is one of the key steps in the biosynthesis pathway of ergosterol (Scheme 5). The protein components of the cytochrome CYP51 system are deeply embedded in the membrane in order to provide a hydrophobic environment that is suitable for the majority of lipophilic substrates to be metabolised [127]. The majority of the cytochrome P-450's are monooxigenases, which means that the substrate is oxidized by insertion of one oxygen atom. The active site of CYP51 contains a ferric prosthetic heme group and after the substrate binds into its binding site protein activation takes place via reduction of the iron atom in the heme system.

Uptake of one oxygen molecule occurs, whereupon a very reactive ferryl oxygen intermediate is formed, which is responsible for the oxidation of the substrate. Azole antifungals form a one-to-one complex with oxidized cytochrome CYP51 inducing a type II spectral change with a peak at about 430 nm (425–430 nm) and a trough at about 398 nm (390–405 nm) [12]. There are indications that azoles also can interact with the reduced form of cytochrome P-450 that means that the uptake of molecular oxygen comes under pressure, which undermines the oxidation of the substrate [128].

The binding of the imidazole and triazole antifungals takes place via coordination of the N3 and N4 of the imidazole and triazole ring respectively with the heme iron of the enzyme. The high affinity of the antifungal azoles not only depends on the azole ring interaction with the heme group but also on the interaction of the lipophilic side chain with the CYP51 apoprotein [127]. At nanomolar concentrations the imidazoles (miconazole, ketoconazole) and the triazoles (terconazole, itraconazole) bind with high affinity to *Candida* CYP51 [126]. Fluconazole, another triazole derivative has a significant lower affinity for the *Candida* CYP51 enzyme. Itraconazole and its imidazole analogue form equistable complexes with *C*.*albicans* CYP51, whereas ketoconazole forms a less stable complex.

Furthermore there is a great selective interaction with the different P-450 enzymes where the constitution of the hydrophobic side chain predominates over the azole ring, which makes it possible to synthesize highly potent and selective P-450 inhibitors [126].

Inhibition of ergosterol biosynthesis has been demonstrated with miconazole, terconazole, ketoconazole, and itraconazole in *C*. *albicans*, *C*. *glabrata*, *C*. *lusitaniae*, *P*. *ovale*, *T*. *mentagrophytes*, *P*. *brasiliensis*, *H*. *capsulatum* and *A*. *fumigatus* [126].

Experiments with purified CYP51 from *S*.*cerevisiae* microsomes demonstrated that three successive oxidation steps are catalysed [12].

Ergosterol is essential for the cell membrane of fungi and is essential for growth of the fungal cell [127]. Inhibition of ergosterol biosynthesis coincides with the accumulation of lanosterol or eburicol. These accumulated lanosterol induces permeability changes, membrane leakage, changes in nutrients transport, and inactivity of membrane bound enzymes, inhibition of growth, increased susceptibility to host-defence mechanisms and eventually cell death [125,126].

Clotrimazole, econazole, ketoconazole, and miconazole inhibited ergosterol biosynthesis in *C. albicans* cells for 80% at 0.1 μM, causing accumulation of methylated sterols, and changing ergosterol/methylated sterol ratio from 20 to 0.2. They also decreased the unsaturated-to-saturated fatty acid ratio from 2.3 to approximately 1.1 [129].

In an *in vivo* experiment *C*. *albicans* cells and the livers were collected from rats with induced vaginal candidiasis after treatment with various oral doses of ketoconazole. In the *Candida* cells, there was a dose dependent shift from ergosterol to lanosterol. This decreased ergosterol biosynthesis, already apparent at 0.63 mg/kg body weight, reached the 50% level at 1.7 mg/kg and was almost complete (100%) at 10 mg/kg. There was a 50% reduction of cholesterol biosynthesis at 10 mg/kg, indicating that at least a six-fold higher dose was required to affect cholesterol biosynthesis in rat liver to the same extent as ergosterol biosynthesis in *C*. *albicans* [130].

The selectivity of ketoconazole and miconazole for fungal CYP51 has been proven in sub-cellular fractions of yeast and mammalian cells. Cholesterol biosynthesis in a sub-cellular fraction from liver is 20–70 times less sensitive to ketoconazole or miconazole than ergosterol biosynthesis in similar fractions from *C*.*albicans* and *Saccharomyces cerevisiae*. Fifty per cent inhibition of cholesterol biosynthesis in intact fibroblast cells was achieved at a ketoconazole concentration of 0.7 μM, whereas only 0.005 μM was needed to give a similar effect on ergosterol biosynthesis in *C*.*albicans* cells incubated in the same medium as the fibroblasts [130].

In fluconazole-susceptible and fluconazole-resistant *C*. *albicans* isolates no significant differences in ergosterol content were found in untreated isolates. Exposure to sub-inhibitory concentrations of voriconazole decreased the total sterol content in *C*. *albicans* isolates and in *C*. *krusei*. Voriconazole was responsible for a complete inhibition of ergosterol biosynthesis and for a 75% reduction in ergosterol content in *C*. *krusei*, whereas an accumulation of ergosterol precursors occurred [84]. In contrast with voriconazole, fluconazole had only a small inhibitory effect on ergosterol biosynthesis with a limited accumulation of ergosterol precursors in all these isolates [84]. Voriconazole is clearly more potent than fluconazole as a CYP51 inhibitor.

The inhibition of sterol biosynthesis in *C*. *albicans* isolates was further evaluated for posaconazole, itraconazole and fluconazole by measuring the IC_50_ values. The IC_50_ values for posaconazole and itraconazole were similar, but the IC_50_ values for fluconazole were 850–7000 times significantly higher [84].

The effect on ergosterol biosynthesis of posaconazole has been studied in *C*. *albicans*, *C*. *glabrata*, *A*. *fumigatus*, and *A*. *flavus*. Ergosterol biosynthesis in all these strains was inhibited in a dose-dependent manner, accompanied by accumulation of eburicol and obtusifoliol, both being methylated precursors of ergosterol [1].

There is evidence that some first generation imidazole antifungals have two distinct antifungal actions. At low concentrations all of them interfere with ergosterol biosynthesis resulting in fungistasis. At higher concentrations (>10 μM) compounds like miconazole, clotrimazole, sulconazole, tioconazole, and sertaconazole can undergo a direct interaction with lipid constituents of the cell membrane in *C*. *albicans* which kills the fungus [61,130,131,132,133].

### 4.2. Antifungal azole drug resistance

The origin of antifungal resistance is multifactorial and results from a combination of circumstances related to the host, the antifungal agent and the pathogen.The immune competence status of the host is an important factor in the occurrence of resistance. Indeed until the late 1980’s, azole resitance was only sporadically reported and always in patients suffering from chronic mucocutaneous candidiasis, who received antifungal therapy over an unusually long period of time. It was the increase in the number of immunocompromised patients, particularly as result of the AIDS pandemic, that preceded the sudden increase of reports on clinical failure to antifungal therapy. Many biochemical studies have been reported to elucidate the antifungal resistance of azoles, for an overview see [176,177,178,179,180,181,182].

In *Candida* cells overexpressing the ERG gene there was a limited effect on MIC of azoles. Moreover such strains are rather rarely found in clinical isolates [125]. On the other hand yeasts or moulds can be resistant to azoles when transmembrane efflux pumps are activated decreasing the intracellular concentration of the drug. In clinical isolates ABC transporters like CDR1 and CDR2 are predominantly involved [125]. Ketoconazole, fluconazole, itraconazole, and voriconazole are good substrates to be transported in contrast with posaconazole which is a relatively poor substrate [125]. In yeasts another family of efflux pumps called MDR1 (earlier called BEN1) is able to transport fluconazole but not posaconazole [125].

In patients with aspergilloma or other cavitary lung diseases caused by *Aspergillus* species and who need treatment for a long period of time azole resistance has been described to emerge in this group of patients. Molecular studies have shown that triazole resistance can be associated with certain amino acid mutations in the CYP51A protein [138].

A phenotype characterised by cross-resistance to itraconazole and posaconazole has been associated with aminoacid substitution at glycine 54 (arginine, glutamate, or tryptophan for glycine), resulting in a 32-fold (arginine and glutamate) to 256-fold (tryptophan) increase in the MIC for posaconazole [125]. A pattern of itraconazole resistance, characterised by different patterns of elevated MIC’s for the other triazole drugs, has been linked to different amino acid substitutions at methionine 220 (M220) [138]. Different mutations in the target enzyme resulting in a lower affinity for azoles have more influence on the antimicrobial activity of fluconazole and voriconazole than of itraconazole or posaconazole [125].

Development of resistance to posaconazole occurs at a low frequency of about 1 in 10^8^ for *A*. *fumigatus*. It is hypothesized that resistance of fungi against posaconazole is associated with mutations in the *CYP 14α-demethylase (CYP51, Erg11p*), resulting in reduced binding [1].

According to Rex *et al*. interpretive breakpoints divide the MIC range into three regions, susceptible (S), susceptible dose-dependent (SDD) and resistant (R) [183]. Despite many *in vitro* studies these breakpoints are, however, only weakly predictive of clinical non-response as summarized by Odds [184] and Rex *et al.* [183]. Indeed in a review article [184] Odds summarized clinical treatment outcomes for fluconazole and itraconazole and it can be concluded that roughly one out of two patients colonized with resistance isolates can still be successfully treated with an azole compound, and that one out of ten patients suffering from infection with susceptible isolate does not respond to this treatment.

### 4.3. Molecular interaction of azoles antifungal agents with CYP51

The binding modes of a series of structurally different imidazoles and triazoles have been studied using molecular modelling techniques. As the crystallographic structure of the cytochrome P-450_14
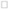
DM_ with azole ligands is not available, the model originally was based on the interaction of cytochrome P‑450_cam_ with 1-phenylimidazole, 2-phenylimidazole, 4-phenylimidazole and metyrapone, respectively. From these data and other data a model has been developed. In this model it became clear that the halogenated phenyl rings were surrounded and oriented to hydrophobic residues. The azole nitrogen, N3 in the imidazoles and N4 in the triazoles coordinates with the heme iron. The etheral oxygen in miconazole and in ketoconazole can undergo a hydrogen bond interaction. Unfortunately the predictions based on docking analysis in this model could not be confirmed by X-ray crystallography [139]. Furthermore a three-dimensional quantitative structure-activity model has been described [140].

Another three-dimensional molecular model of the fungal form of cytochrome P450 (CYP51) derived from *S. cerevisiae*, based on homology with the haemoprotein domain of CYP102 from *Bacillus megaterium* (a unique bacterial P450 with a known crystal structure) is described [115]. In this model lanosterol can readily occupy the binding site in such a way, that lanosterol 14α-demethylation is the preferred route of metabolism. Key interactions between the substrate and amino acid side chain in the binding site appear to orientate lanosterol in such a way that the oxidation at the C_14_-methyl group can readily occur [141]. In this model azole inhibitors like ketoconazole are able to fit into the putative binding site of CYP51 by an interaction combination of heme ligation, hydrogen bonding, π−π stacking interactions between aromatic rings of the inhibitor and protein, and hydrophobic interactions within the heme environment of the enzyme. The mode of action of azole antifungals, as described by modelling studies, is supported by quantitative structure-activity relationship (QSAR) analyses on two groups of structurally related fungal inhibitors [141]. We have to keep in mind that the modelling is based on a *S*. *cerevisiae* P-450 model and the QSAR study is based on activity in *C*. *albicans*, which makes things more complicated.

## 5. Drug Interactions in the Clinic

Cytochrome P-450s play an important role in the metabolism of sterols, steroids, bile acids, vitamin A and D, thromboxane A_2_, prostacyclin, and leukotrienes. Furthermore they catalyse the metabolism of xenobiotics [126]. The antifungal azoles are metabolized by CYP3A4, in addition the interaction of the imidazole or triazole moiety interacts with the CYP3A4 heme group and causes moderate to strong inhibition of CYP3A4 [173]. This means that all of them potentially can interact with the metabolism of other drugs and potentiall enhance the concentration of xenobiotics. On the other hand drugs inducing CYP3A4 can be responsible for lower concentrations of antifungal drugs: e.g., drugs like rifampin, carbamazepine, ritonavir, efavirenz, or rifabutin decrease triazole plasma concentrations [107].

Secondary effects of azoles were possibly drug-specific, such as inhibition of cytochrome-c peroxidase and catalase leading to the accumulation of hydrogen peroxide [134], blocking of electron transport in the respiratory chain [135] and inhibition of hyphae formation [72].

In the biochemical transformation of drugs microsomal drug metabolising enzymes such as some cytochrome P-450s play an important role in drug metabolism. Liver homogenates and subcellular preparations have been used to study the various steps in the metabolism of a drug. The antifungal activity depends on the inhibition of fungal cytochrome P-450, which makes it necessary to study the possible interference of azole antifungal drugs with metabolism patterns of other drugs in combination therapy [136].

Drug-drug interactions with azoles are numerous and it would go much beyond the aim of this review to exemplify these drug-drug interactions. Although we want to include a few examples for a more indepth expert view on this topic we refer to the following references [1,88,107,142,173,185,186,188]. Ketoconazole for example markedly increased blood concentrations of everolimus [88].

Fluconazole exerts a considerable inhibitory activity on CYP2C9 and CYP3A4 at lower and higher daily dosages, respectively [88].

Itraconazole (fluconazole) has a marked inhibitory effect on CYP3A4 and some antiarrhytmic CYP3A4 substrates like quinidine and dofelide are contraindicated. Itraconazole also interferes with vincristine metabolism. Furthermore itraconazole has an inhibitory effect on busulfan degradation, which depends on glutathione-*S*-transferase activity [88].

Voriconazole is a potent inhibitor of several cytochrome P-450 isozymes including CYP2C9, CYP2C19 and CYP3A4 and as a consequence the spectrum of observed drug-drug interactions is continuously increasing and is even more pronounced than that of itraconazole. In particular the extent of interaction may sometimes be higher than expected when the co-administered drug undergoes an extensive first-pass effect through the stomach and the liver (for example, simvastatin, tacrolimus, and sirolimus) [88]. Voriconazole is primarily metabolised by CYP2C19 and CYP3A4 [84,108]. Therefore plasma concentration can be affected by co-administrated drugs that modulate CYP2C19 or CYP3A4 activities [142].

Similar to itraconazole and voriconazole, posaconazole is a potent CYP3A4 inhibitor. The interaction is more pronounced when posaconazole and the co-administered drug are given orally and when the co-administered drug undergoes an extensive first-pass effect [88].

Posaconazole has no significant effect on the activity of CYP2C8/9, CYP1A2, CYP2D6, or CYP2E1 [1]. Tacrolimus which is metabolized by CYP3A4, exposure is significantly increased after co-administration with posaconazole. The dose of cyclosporine has to be reduced up to 28.6% after coadministration with posaconazole in heart transplant patients [1].

Co-administration of cimetidine and posaconazole has resulted in an about 40% reduction in AUC and C_max_ values for posaconazole [1]. Triazole antifungals should be administered with caution to patients taking cytochrome P4503A4 substrates [107].

Moreover triazoles increase plasma concentrations of sirolimus and ergot alkaloids and should not be used in combination, especially when drug levels are not monitored [107].

In drug-drug interactions we have to keep in mind that P-glycoprotein, an efflux protein is extensively co-localized with CYP3A4 in the intestine, the liver, the kidneys and the cells of the blood-brain barrier. Itraconazole is an inhibitor and substrate of P-glycoprotein, but voriconazole is neither a substrate nor an inhibitor. Given the structural similarity between itraconazole and posaconazole, the latter probably is a P-glycoprotein substrate and inhibitor [84,187].

## 6. Conclusions

**Figure 9:**
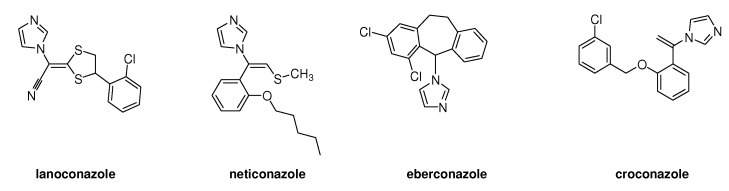
Topical non-miconazole-like conazoles.

In the antimycotic conazoles there has been a steady evolution from agents which only could be utilised for topical applications with miconazole and related compounds as examples. Some other novel antimycotics such as lanoconazole, eberconazole croconazole, and neticonazole are topically applied antimycotics and are imidazole derivatives. All of them display similar activity compared to the other topically applied drugs (Figure 9) [146,147,148,149].

After the discovery of ketoconazole the era of orally available conazoles was heralded with on the one hand itraconazole and posaconazole which are structurally closely related and on the other hand the closely related compounds fluconazole and voriconazole. The first generation was mainly used to treat superficial mycoses and the second generation can be applied for treatment of emerging invasive fungal infections.The need for more powerful drugs to treat invasive fungal infections is of high importance [143,150,151,152].

**Figure 10:**
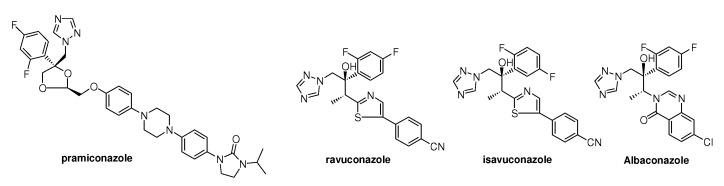
Emerging conazoles under clinical evaluation for systemic fungal infections.

New potential conazoles like albaconazole [153,154,155] and ravuconazole [156,157], isavuconazole [158,159,160] and pramiconazole [161,162,163,164] and other potential more potent conazoles with a more favorable side-effect profile deserve to be developed (Figure 10). In contrast to many other classes like the allylamines [165,166], the echinocandins [167,168], the sordanines [169,170] and polyenes the conazoles have a broad-spectrum activity against many yeasts and moulds. Notwithstanding that the antifungal spectrum is narrower than for the conazoles, some members of the new non-azole classes are needed for treatment of aggressive invasive fungal infections.

## References

[1] Schiller D.S., Fung H.B. (2007). Posaconazole: An Extended- Spectrum Triazole Antifungal. Clin. Ther..

[2] Godefroi E.F., Heeres J., Van Cutsem J., Janssen P.A.J. (1969). The Preparation and Antimycotic Properties of Derivatives of 1-Phenethylimidazole. J. Med. Chem..

[3] Godefroi E.F. (1968). Reductive ring cleavage of 1,3-disubstituted imidazolium iodides by sodium borohydride. J. Org. Chem..

[4] Tajana A., Sibilia C., Cappelletti R., Cova A., Nardi D. (1981). Physico-Chemical, Structural and Analytical Studies on Fenticonazole, a New Drug with Antimycotic Properties. Arzneim.-Forsch./Drug Res..

[5] Heeres J., Backx L.J.J., Van Cutsem J. (1977). Antimycotic Imidazoles. 3. Synthesis and Antimycotic Properties of 1-[2-(Aryloxyalkyl)-2-phenylethyl]-1*H*-imidazoles. J. Med. Chem..

[6] Mixich G., Thiele K. (1979). Ein Beitrag zur stereospezifischen Synthese von antimykotisch wirksamen Imidazolyloximäthern. Arzneim.-Forsch./Drug Res..

[7] Walker K.A.M., Braemer A.C., Hitt S., Jones R.E., Matthews T.R. (1978). 1-[4-(4-Chlorophenyl)-2-(2,6-dichlorophenylthio)-*n*-butyl]-1*H*-imidazole Nitrate, a New Potent Antifungal Agent. J. Med. Chem..

[8] Heeres J., Van Cutsem J. (1981). Imidazoles, 5, Synthesis and Antimycotic Properties of 1-[[2-Aryl-4(aralkyl)-1,3-dioxolan-2-yl]methyl]-1*H*-imidazoles. J. Med. Chem..

[9] Heeres J., Backx L.J.J., Van Cutsem J. (1979). Antimycotic Imidazoles. Part 4. Synthesis and Antifungal Activity of Ketoconazole, a Potent Orally Active Broad-Spectrum Antifungal Agent. J. Med.Chem..

[10] Heeres J., Hendrickx R., Van Cutsem J. (1983). Antimycotic Azoles, 6, Synthesis and Antifungal Properties of Terconazole, a Novel Triazole Ketal. J. Med. Chem..

[11] Heeres J., Backx L.J.J., Van Cutsem J. (1984). Antimycotic Azoles. 7. Synthesis and Antifungal Properties of a Series of Novel Triazol-3-ones. J. Med. Chem..

[12] Vanden Bossche H., Heeres J., Backx L.J.J., Marichal P., Willemsens G., Rippon J.W., Fromtling R.A. (1993). Discovery, Chemistry, Mode of Action, and Selectivity of Itraconazole. Cutaneus Antifungal Agents.

[13] Saksena A.K., Girijavallabhan V.M., Wang H., Liu Y.-T., Pike R.E., Ganguly A.K. (1996). Consise Asymmetric routes to 2,2,4-trisubstituted tetrahydrofurans via chiral titanium imide enolates: key intermediates towards synthesis of highly active azole antifungals Sch 51048 and Sch 56592. Tetrahedron Lett..

[14] Saksena A.K., Girijavallabhan V.M., Lovery R.G., Pike R.E., Wang H., Liu Y.T., Pinto P., Bennett F., Jao E., Patel N., Desai J.A., Rane D.F., Cooper A.B., Ganguly A.H., Bently P.H., O’Hanlon P.J. (1997). Advances in the Chemistry of Novel Broad-Spectrum Orally Active Azole Antifungals: Recent Studies Leading to the Discovery of Sch 56592. Anti-Infectives Recent Advances in Chemistry and Structure-Activity Relationships.

[15] Hitchcock C.A., Whittle P.J., Rippon E.J.W., Fromtling R.A. (1993). Chemistry and Mode of Action of Fluconazole. Cutaneus Antifungal Agents.

[16] Butters M., Ebbs J., Green S.P., MacRae J., Morland M.C., Murtiashaw C.W., Pettman A.J. (2001). Process Development of Voriconazole: A Novel Broad-Spectrum Triazole Antifungal agent. Org. Process Res. Dev..

[17] Jevons S., Gymer G.E., Brammer K.W., Cox D.A., Leeming M.R.G. (1979). Antifungal Activity of Tioconazole (UK-20,349), a New Imidazole Derivative. Antimicrob. Agents Chemother..

[18] Veronese M., Salvaterra M., Barzaghi D. (1981). Fenticonazole, a New Imidazole Derivative with Antibacterial and Antifungal Activity. Arzneim.-Forsch./Drug Res..

[19] Graziani G., Cazzulani P., Barbadoro B. (1981). Toxicological and Pharmacological Properties of Fenticonazole, a New Topical Antimycotic. Arzneim.-Forsch./Drug Res..

[20] Costa A.L. (1982). “*In vitro*” Antimycotic Activity of Fenticonazole (Rec 15/1476). Mykosen.

[21] Veronese M. (1984). Fenticonazole: A New Antifungal Imidazole Derivative *In vitro* and *In vivo* Activity. Mykosen.

[22] Veronese M., Barzaghi D., Bertonccini A. (1981). Antifungal activity of Fenticonazole in Experimental Dermatomycosis and Candidiasis. Arzneim.-Forsch./Drug Res..

[23] Yoshida H., Kasuga O., Yamaguchi T. (1984). Studies on Antifungal Activities of Sulconazole. Chemotherapy.

[24] Ortiz J.A.  (1992). Sertaconazole, A New Antifungal agent. Arzneim.-Forsch./Drug Res..

[25] Palacin C., Tarrago C., Agut J., Guglietta A. (2001). *In vitro* Activity of Sertaconazole, Ketoconazole, Fenticonazole, Clotrimazole and Itraconazole Against Pathogenic Vaginal Yeast Infections, Methods Find. Exp. Clin. Pharmacol..

[26] Rotstein D.M., Walker K.A.M. (1993). The Synthesis and Antifungal Activity of the Enantiomers of Butoconazole Nitrate. Tetrahedron Assymmetry.

[27] Heeres J. New Azole Compounds: From Hypnotics to Itraconazole, a Broad-Spectrum Antifungal Agent. Actualités Chim. Therapeut; Proceedings of 28^emes^ Rencontre Internationales de Chimie Therapéutique.

[28] Thienpont D., Van Cutsem J., Van Gerven F., Heeres J., Janssen P.A.J. (1979). Ketoconazole - a new broad-spectrum orally active antimycotic. Experientia.

[29] Richardson K., Rippon J.W., Fromtling R.A. (1993). The Discovery of Fluconazole. Cutaneus Antifungal Agents.

[30] Dickinson R.P., Bell A.S., Hitchcock C.A., Narayanaswami S., Ray S.J., Richardson K., Troke P.F. (1996). Novel antifungal 2-aryl-1-(1H-1,2,4-triazol-1-yl)butan-2-ol derivatives with high activity against *Aspergillus fumigatus*. Bioorg. Med. Chem. Lett..

[31] Van Cutsem J.M., Thienpont D. (1972). Miconazole, a Broad-Spectrum Antimycotic Agent with Antibacterial activity. Chemotherapy.

[32] Heel R.C., Brogden R.N., Pakes G.E., Speight T.M., Avery G.S. (1980). Miconazole: A Preliminary Review of its Therapeutic Efficacy In Systemic Fungal Infections. Drugs.

[33] Hernandez Molina J.M., Llosa J., Martinez Brocal A., Ventosa A. (1992). *In vitro* activity of cloconazole, sulconazole, butoconazole, isoconazole, fenticonazole, and five other antifungal agents against clinical isolates of *Candida albicans* and *Candida* spp.. Mycopathalogia.

[34] Odds F.C., Cheesman A.B.C.L., Abbott A.B. (1985). Suppression of ATP in *Candida albicans* by imidazole and derivative antifungal agents. Sabouraudi: J. Med. Vet. Mycol..

[35] Brugmans J., Van Cutsem J., Heykants J., Schuurmans V., Thienpont D. (1972). Systemic antifungal potential, safety, biotransport and transformation of miconazole nitrate. Eur. J. Clin. Pharmacol..

[36] Lewi P.J., Boelaert J., Daneels R., De Meyere R., Van Landuyt H., Heykants J., Symoens J., Weynants J. (1976). Pharmacokinetic profile of intravenous miconazole in man. Comparison of normal subjects and patients with renal insufficiency. Eur. J. Clin. Pharmacol..

[37] Janssen P.A.J., Van Bever W.F.M. (1979). Miconazole. Pharmacological and Biochemical Properties of Drug Substances.

[38] Thienpont D., Van Cutsem J., Van Nueten J.M., Niemegeers C.J.E., Marsboom R. (1975). Biological and toxicological properties of econazole, a broad-spectrum antimycotic. Arzneim.-Forsch..

[39] Levine H.B. (1976). R34000, a dioxolane imidazole in the therapy for experimental coccidioidomycosis. Chest.

[40] Cartwright R.Y. (1978). Absorbtion of econazole from the human gastrointestinal tract. Curr. Chemothery: Proceedings of International Congress Chemotherapy.

[41] Heel R.C., Brogden R.N., Speight T.M., Avery G.S. (1978). Econazole: A Review of its Antifungal Activity and Therapeutic Efficacy. Drugs.

[42] Aron-Brunetiere R., Dompmartin-Pernot D., Drouhet E. (1977). Treatment of pityriasis capitis (dandruff) with econazole nitrate. Acta Dermatovener.

[43] Kessler H.J. (1979). Mikrobiologische Untersuchungen mit Isoconazolnitrat, einem Breitspectrum-Antimykoticum aus der Gruppe der Imidazol-Derivate. Arzneim.-Forsch./Drug Res..

[44] Fromtling R.A. (1988). Overview of Medically Important Azole Derivatives. Clin. Microbiol. Rev..

[45] Fark B., Földes M., Kajtar I. (1982). Results of Isoconazol Nitrate Treatment of Vaginal Mycoses. Mykosen.

[46] Herms E., Kallischnigg G. (1988). Once-Daily-Application of Isoconazole as Cream, Solution and Spray: Comparative Studies on Patients with Dermatomycoses. Z. Hautkr..

[47] Weitgasser H., Herms E. (1979). Comparative Clinical Investigations with the New Antimycotic Agent Isoconazole Nitrate and its Combination with Diflucortolone-21-valerate in the Case of Inflammatory and Eczematised Dermatomycoses. Mykosen.

[48] Oyeka C.A., Gugnani H.C. (1992). Isoconazole Nitrate versus clotrimazole in foot and nail infections due to *Hendersonula toruloidea*, *Scytalidium hyalinum* and dermatophytes. Mycoses.

[49] Eschborn H.M. (1993). Fenticinazole. A local antimycotic. Mykosen.

[50] Polak A. (1982). Oxiconazole, a New Imidazole Derivative. Arzneim.-Forsch./Drug Res..

[51] Gebhart R.J., Espinel-Ingroff A., Shadomy S. (1984). *In vitro* Susceptibility Studies with Oxiconazole. Chemotherapy.

[52] Jegasothy B.V., Pakes G.E. (1991). Oxiconazole Nitrate: Pharmacology, Efficacy, and Safety of a New Imidazole Antifungal Agent. Clin. Ther..

[53] Wagner W. (1986). Vergleich der klinischen Wirksamkeit und Verträglichkeit von Oxiconazol nach 1x täglicher Applikation mit der nach 2x täglicher Application. Mykosen.

[54] Odds F.C. (1980). Laboratory evaluation of antifungal agents: a comparative study of five imidazole derivatives of clinical importance. J. Antimicrob. Chemother..

[55] Clissold S.P., Heel R.C. (1986). Tioconazole, A Review of its Antimicrobial Activity and Therapeutic Use in Superficial Mycoses. Drugs.

[56] Krohn K., Vinnerberg A. (1983). Open Comparison of the Efficacy, Toleration and Safety of Tioconazole and Econazole in the 3-Day Treatment of Vaginal Candidiasis. Gynäk. Rdsch..

[57] Artner J., Fuchs G. (1983). Open Studies of the Efficacy, Tolerance, Systemic Absorbtion and Vaginal Persistence following a Single Application of Tioconazole Ointment in the Treatment of Patients with vaginal Candidosis. Gynäk. Rdsch..

[58] Clayton Y.M., Hay R.J., McGibbon D.H., Pye R.J. (1982). Double Blind comparison of the efficacy of tioconazole and miconazole for the treatment of fungal infection of the skin orerythrasma. Clin. Exp. Dermatology.

[59] Seidman L.S., Skokos C.K. (2005). An evaluation of butoconazole nitrate 2% Site Release vaginal cream (Gynazole-1) compared to fluconazole 150 mg tablets (Diflucan) in the time to releif of symptoms in patients with vulvovaginal candidiasis. Infect. Dis. Obstet. Gynecol..

[60] Palacin C., Sacristan A., Ortiz J.A. (1992). *In vitro* Activity of Sertaconazole. Arzneim.-Forsch./Drug Res..

[61] Carillo-Munoz A.J., Giusiano G., Ezkurra P.A., Quindos G. (2005). Sertaconazole: updated review of a topical antifungal agent. Expert Rev. Anti Infect. Ther..

[62] Palacin C., Sacristan A., Ortiz J.A. (1992). *In vivo* Activity of Sertaconazole in Experimental Dermatophytosis in Guinea Pigs. Arzneim.-Forsch./Drug Res..

[63] Pedragosa R., Gonzalez B., Martin M., Herrero E., Roset P., Marquez M., Torres J., Ortiz J.A. (1992). Therapeutic Efficacy and Safety of the New Antimycotic Sertaconazole in the Treatment of Pityriasis versicolor. Arzneim.-Forsch./Drug Res..

[64] Van Cutsem J., Van Gerven F., Zaman R., Janssen P.A.J. (1983). Terconazole, a new broad-spectrum antifungal. Chemotherapy.

[65] Tolman E.I., Isaacson D.M., Rosenthale M.E., McGuire J.L., Van Cutsem J., Borgers M., Vanden Bossche H. (1986). Anticandidal Activities of Terconazole, a Broad-Spectrum Antimycotic. J. Antimicrob. Chemother..

[66] Tolman E.L., Isaacson D.M., Rosenthale M.E. (1985). *In vitro* Studies with Terconazole. Gynäk. Rdsch..

[67] Hirsch H.A. (1989). Clinical Evaluation of Terconazole. European Experience. J. Reprod. Med..

[68] Cauwenbergh G. (1989). Terconazole. Pharmacology of a new antimycotic agent. J. Reprod. Med..

[69] Sood G., Nyirjesy P., Velma Weitz M., Chatwani A. (2000). Terconazole cream for Non-*Candida Albicans* Fungal vaginitis: Results of a Retrospective Analysis. Infect. Dis. Obstet. Gynecol..

[70] Raab W. (1985). Terconazole, ein neues Triazolderivaat zur Behandlung vaginaler Mykosen. Gynäk. Rdsch..

[71] Heel R.C., Brogden R.N., Carmine A., Morley P.A., Speight T.M., Avery G.S. (1982). Ketoconazole: A Review of its Therapeutic Efficacy in Superficial Fungal Infections. Drugs.

[72] De Brabander M., Aerts F., Van Cutsem J., Vanden Bossche H., Borgers M. (1980). The activity of ketoconazole in mixed cultures of leukocytes and Candida albicans. Sabouraudia.

[73] Strippoli V., Piacentini A., D’Auria F.D., Simonetti N. (1997). Antifungal Activity of Ketoconazole and Other Azoles against *Mallassezia furfur In vitro* and* In vivo*. Infection.

[74] Borelli P., Bran J.L., do Fuentes J., Legendre R., Leiderman E., Levine H.B., Restrepo A., Stevens D.A. (1979). Ketoconazole, an oral antifungal: laboratory and clinical assessment of imidazole drugs. Postgrad. Med. J..

[75] Levine H B., Cobb J.M. (1978). Oral therapy for experimental coccidiodomycosis with R41400 (ketoconazole), a new imidazole. Am. Rev. Respir. Dis..

[76] Williams D.M., Graybill J.R., Drutz D.J., Levine H.J. (1980). Suppression of Cryptococcosis and Histoplasmosis by Ketoconazole in Athymic Nude Mice. J. Infect. Dis..

[77] Huang Y.-C., Colaizzi J., Bierman R.H., Woestenborghs R., Heykants J. (1986). Pharmacokinetics and Dose Proportionality of Ketoconazole in Normal Volunteers. Antimicrob. Agents Chemother..

[78] Gascoigne E.W., Barton G.J., Michaels M., Meuldermans W., Heykants J. (1981). The kinetics of ketoconazole in animals and man. Clin. Res. Rev..

[79] Van Tyle J.H. (1984). Drugs in Perspective, Ketoconazole, Mechanism of Action, Spectrum of Activity, Pharmacokinetics, Drug Interactions, Adverse Reactions and Therapeutic Use.. Pharmacotherapy.

[80] Rosenblatt H.M., Byrne W., Ament M.E., Graybill J., Stiehm E.R. (1980). Successful treatment of chronic mucocuteneous candidiasis with ketoconazole. J. Pediat..

[81] Smith E.B., Henry J.C. (1984). Ketoconazole: An Orally Efective Antifungal Agent, Mechanism of Action, Pharmacology, Clinical Efficacy and Adverse Effects. Pharmacotherapy.

[82] Calhoun D.l., Waskin H., White M.P., Bonner J.R., Mulholland J.H., Rumans L.W., Stevens D.A., Galgiani J.N. (1991). Treatment of Systemic Sporotrichosis with Ketoconazole. Rev. Infect. Dis..

[83] Van Cutsem J., Van Gerven F., Zaman R., Heeres J., Janssen P.A.J. (1983). Pharmacological and preclinical results with a new oral and topical broad-spectrum antifungal, R51211. Processdings of 13th International Congress of Chemotherapy.

[84] Hoffman H.L., Ernst E.J., Klepser M.E. (2000). Novel triazole antifungal agents. Exp. Opin. Invest. Drugs.

[85] Espinel-Ingroff A., Shadomy S., Gebhart R.J. (1984). *In vitro* studies with R 51,211. Antimicrob. Agents Chemother..

[86] Borgers M., Van de Ven M.-A. (1987). Degenerative changes in fungi after itraconazole treatment. Rev. Infect. Dis..

[87] Van Cutsem J., Van Gerven F. (1986). The *In vivo* Antifungal Activity of Broad-Spectrum Azoles. Drug Dev. Res..

[88] Lipp H.-P. (2008). Antifungal agents-clinical pharmacokinetics and drug interactions. Mycoses.

[89] Gubbins P.O. (2007). Mould-active azoles: pharmacokinetics, drug interactions in neutropenic patients. Curr. Opin. Infect. Dis..

[90] Kunze K.L., Nelson W.L., Karasch E.D., Thummel K.E., Isoherranen N. (2006). Stereochemical aspects of itraconazole metabolism *in vitro* and *in vivo*. Drug Metab. Dispos..

[91] Degreef H.J., De Doncker P.R.G. (1994). Current therapy of dermatophytosis. J. Am. Acad. Dermatol..

[92] De Doncker P., Van Lint J., Dockx P., Roseeuw D. (1995). Pulse therapy with one-week itraconazole monthly for three or four months in the treatment of onychomycosis. Cutis.

[93] Cauwenbergh G. (1986). New and prospective developments in antifungal drugs. Acta Derm. Venereol. Suppl. (Stockh.).

[94] Battegay M., Flückiger U. (2003). Therapy schwerer Pilzinfectionen. Internist.

[95] Kauffman C. A. (1994). Newer developments in therapy for endemic mycoses. Clin. Infect. Dis..

[96] Graybill J.R. (2000). Changing Strategies for Treatment of Systemic Mycoses. Braz. J. Infect. Dis..

[97] Hughes C.E., Beggs W.H. (1987). Action of fluconazole (UK-49,858) in relation to other systemic antifungal agents. J. Antimicrob. Chemother..

[98] Odds F.C., Cheesman S.L., Abbott A.B. (1986). Antifungal effects of fluconazole (UK 49,858), a new triazole antifungal, *in vitro*. J. Antimicrob. Chemother..

[99] Odds F.C., Webster C.E. (1988). Effects of azole antifungals *in vitro* on host/parasite interactions relevant to candida infections. J. Antimicrob. Chemother..

[100] Nguyen M.H., Yu C. (1998). Voriconazole against fluconazole-susceptible and –resistant *Candida* isolates: *in vitro* efficacy compared with that of itraconazole and ketoconazole. J. Antimicrob. Chemother..

[101] Nguyen M.H., Yu C. (1998). *In vitro* comparative efficacy of voriconazole and itraconazole against fluconazole-susceptible and -resistant *Cryptococcus neoformans* isolates. Antimicrob. Agents Chemother..

[102] Classen D.C., Burke J.P., Smith C.B. (1988). Treatment of Coccidioidal Meningitis with Fluconazole. J. Infect. Dis..

[103] Nagappan V., Deresinsky S. (2007). Posaconazole: A Broad-Spectrum Triazole Antifungal agent. Clin. Infect. Dis..

[104] Manavathu E.K., Abraham O.C., Chandrasekar P.H. (2001). Isolation of *in vitro* susceptibility to amphotericin B, itraconazole and posaconazole of voriconazole-resistant laboratory isolates of *Aspergillus fumigatus*. Clin. Microb. Infect..

[105] Koltin Y., Hitchcock C.A. (1997). The search for new triazole antifungal agents. Curr. Opin. Chem. Biol..

[106] Gigolashvili T. (1999). Update on Antifungal Therapy. Cancer Pract..

[107] Aperis G., Mylonakis E. (2006). Newer triazole antifungal agents: pharmacology, spectrum, clinical efficacy and limitations. Expert Opin. Invest. Drugs.

[108] Dupont B. (2003). Nouveaux antifongiques: voriconazole et caspofungine. Arch. de pédiatrie.

[109] Barchiesi F., Arzeni D., Camiletti V., Simonetti O., Cellini A., Offidani A.M., Scalise G. (2001). *In vitro* Activity of Posaconazole against Clinical Isolates. J. Clin. Microbiol..

[110] Marco F., Pfaller M.A., Messer S.A., Jones R.N. (1998). *In vitro* activity of a new triazole antifungal agent, Sch 56592, against clinical isolates of filamentous fungi. Mycopathologia.

[111] Sugar A.M., Liu X.-P. (1996). *In vitro* and *In vivo* Activities of SCH 56592 against *Blastomyces dermatitidis*. Antimicrob. Agents Chemother..

[112] Lutz J.E., Clemons K.V., Aristizabal B.H., Stevens D.A. (1997). Activity of the Triazole SCH 56592 against Disseminated Murine Coccidiodomycosis. Antimicrob. Agents Chemother..

[113] Connolly P., Wheat L.J., Schizlein-Bick C., Durkin M., Kohler S., Smedema M., Goldberg J., Brizendine E., Loebenberg D. (2000). Comparison of a New triazole, Posaconazole with Itraconazole and Amphotericin B for Treatment of Histoplasmosis following Pulmonary Challenge in Immunocompromised Mice. Antimicrob. Agents Chemother..

[114] Connolly P., Wheat J., Schizlein-Bick C., Durkin M., Kohler S., Smedema M., Goldberg J., Brizendine E., Loebenberg D. (1999). Comparison of a New triazole Antifungal agent, Schering 56592 with Itraconazole and Amphotericin B for Treatment of Histoplasmosis in Immunocompetent Mice. Antimicrob. Agents Chemother..

[115] Cacciapuoti A., Loebenberg D., Corcoran E., Menzel F., Moss E.L., Norris C., Michalski M., Raynor K., Halpern J., Mendrick C., Arnold B., Antonacci B., Parmegiani R., Yarosh-Tomaine T., Miller G.H., Hare R.S. (2000). *In vitro* and *In vivo* Activities of SCH 56592 (Posaconazole), a New Triazole Antifungal Agent, against *Aspergillus* and *Candida*. Antimicrob. Agents Chemother..

[116] Oakley K.L., Morrissey G., Denning D.W. (1997). Efficacy of SCH-56592 in a Temporariy Neutropenic Murine Model of Invasive Aspergillosis with an Itraconazole-susceptible and Itraconazole-Resistant Isolate of *Aspergillus fumigatus*.. Antimicrob. Agents Chemother..

[117] Graybill J.R., Bocanegra R., Najvar L.K., Luther M.F., Loebenberg D. (1998). SCH56592 treatment of murine invasive aspergillosis. J. Antimicrob. Chemother..

[118] Lozano-Chiu M., Arikan S., Paetznick V.L., Anaissie E.J., Loebenberg D., Rex J.H. (1999). Treatment of Murine Fusariosis with SCH 56592. Antimicrob. Agents Chemother..

[119] Nomeir A.A., Kumari P., Hilbert M.J., Gupta S., Loebenberg D., Cacciapuoti A., Hare R., Miller G.H., Lin C.C., Cayen M.N. (2000). Pharmacokinetics of SCH 56592, a New Azole Broad-Spectrum Antifungal Agent, in Mice, Rats, Rabbits, Dogs, and Cynomolgus Monkeys.. Antimicrob. Agents Chemother..

[120] Sansone-Parsons A., Krishna G., Calzetta A., Wexler D., Kantesaria B., Rosenberg M.A., Saltzman M.A. (2006). Effect of a Nutritional Supplement on Posaconazolez Pharmacokinetics following Oral Administration to Healthy Volunteers. Antimicrob. Agents Chemother..

[121] Chhun S., Rey E., Tran A., Lortholary O., Pons G., Jullien V. (2007). Simultaneous quantification of voriconazole and posaconazole in human plasma by high-performance liquid chromatography with ultra-violet detection. J. Chromatogr. B.

[122] Stevens D.A., Rendon A., Gaona-Flores V., Catanzaro A., Anstead G.M., Pedicone L., Graybill J.R. (2007). Posaconazole Therapy for Chronic Refractory Coccidiodomycosis. Chest.

[123] Walsh T.J., Raad I., Patterson T.F., Chandrasekar P., Donowitz G.R., Graybill J.R., Greene R.E., Hachem R., Hadley S., Herbrecht R., Langston A., Louie A., Ribaud P., Segal B.H., Stevens D.A., van Burik J.-A.H., White C.S., Corcoran G., Gogate J., Krishna G., Pedicone L., Hardalo C., Perfect J.R. (2007). Treatment of Invasive Aspergillosis with Posaconazole in Patients Who Are Refractory to or Intolerant of Conventional Therapy: An Externally Controlled Trial. Clin. Infect. Dis..

[124] Raad I.I., Graybill J.R., Bustamante A.B., Cornely O.A., Gaona-Flores V., Afit C., Graham D.R., Greenberg R.N., Hadley S., Langston A., Negroni R., Perfect J.R., Pitisuttithum P., Restrepo A., Schiller G., Pediconi L., Ullmann A.J. (2006). Safety of long-term oral posaconazole use in the treatment of refractory invasive fungal infections. Clin. Infect. Dis..

[125] Hof H. (2006). A new, broad-spectrum azole antifungal: posaconazole- mechanism of action and resistance, spectrum of activity. Mycoses.

[126] Vanden Bossche H., Marichal P. (1991). Mode of action of anti-*Candida* drugs: Focus on terconazole and other ergosterol biosynthesis inhibitors. Am. J. Obstet. Gynecol..

[127] Boiron P. (1995). Azole Antifungals. Infect. Dis. Ther..

[128] Vanden Bossche H., McGinnis M.R. (1985). Biochemical targets for antifungal azole derivatives: hypothesis on the mode of action. Current Topics in Medical Mycology.

[129] Georgopapadakou N.H., Dix B.A., Smith S.A., Freudenberger J., Funke P.T. (1987). Effect of Antifungal Agents on Lipid Biosynthesis and Membrane Integraty in *Candida albicans*. Antimicrob. Agents Chemother..

[130] Vanden Bossche H., Lauwers W., Willemsens G., Marichal P., Cornelissen F, Cools W. (1984). Molecular Basis for the Antimycotic and Antibacterial Activity of N-Substituted Imidazoles and Triazoles: the Inhibition of Isoprenoid Biosynthesis. Pestic. Sci..

[131] Sud J.J., Feingold D.S. (1981). Mechanism of action of the antimycotic imidazoles. J. Invest. Dermatol..

[132] Beggs W.H. (1994). Mechanism of the rapid methal action of miconazole and sulconazole against *Candida albicans*. Biochem. Arch..

[133] Beggs W.H. (1987). Rapid fungicidal action of tioconazole and miconazole. Mycopathologica.

[134] De Nollin S., Van Belle H., Goossens F., Thone F., Borgers M. (1977). Cytochemical and biochemical studies of yeasts after *in vitro* exposure to miconazole. Antimicrob. Agents Chemother..

[135] Shigematsu M.L., Arai T. (1982). Effect of ketoconazole on isolated mitochondria from *Candida albicans*. Antimicrob. Agents Chemother..

[136] Niemegeers C.J.E., Levron J.C., Awouters F., Janssen P.A.J. (1981). Inhibition and Induction of Microsomal Enzymes in the Rat. A Comparative Study of Four Antimycotics: Miconazole, Econazole, Clotrimazole and Ketoconazole. Arch. Int. Pharmacodyn..

[137] Pfaller M.A., Riley J., Koerner T. (1990). Effects of Terconazole and Other Azole Antifungal Agents on the Sterol and Carbohydrate Composition of *Candida Albicans*. Diagn. Microbiol. Infect. Dis..

[138] Verweij P.E., Snelders E., Melchers W.J.G., Varga J., Samson R.A. (2008). Azole resistance in Aspergillus fumigatus. Aspergillus in the Genomic Era.

[139] Talele T.T., Hariprasad V., Kulkarni V.M. (1998). Docking Analysis of a Series of Cytochrome P-450_14αDM_ Inhibiting Azole Antifungals. Drug Des. Discov..

[140] Tatele T.T., Kulkarni V.M. (1999). Three-Dimensional Quantitative Structure-Activity Relationship (QSAR) and Receptor Mapping of Cytochrome P-450_14αDM_ Inhibiting Azole Antifungal Agents. J. Chem. Inf. Comput. Sci..

[141] Lewis D.F.V., Wiseman A., Tarbit M.H. (1999). Molecular modeling of lanosterol 14α-demethylase (CYP51) from *Sacharomyces cerevisiae* via homology with CYP102, a unique bacterial cytochrome P450 isoform: Quantitative Structure-Activity Relationships (QSARs) within two related series of antifungal azole derivatives. J. Enzyme Inhib..

[142] Johnston A. (2003). The pharmacokinetics of voriconazole. Br. J. Clin. Pharmacol..

[143] Chiou C.C., Groll A.H., Walsh T.J. (2000). New Drugs and Novel Targets for Treatment of Invasive Fungal Infections in Patients with Cancer. The Oncologist.

[144] Arias A., Arevalo M.P., Adreu A., Rodriguez C., Sierra A. (1996). *Candida glabrata*: *In vitro* susceptibility of 84 isolates to Eight Antifungal Agents. Chemotherapy.

[145] Niwano Y., Tabuchi T., Kanai K., Hamaguchi H., Uchida K., Yamaguchi H. (1995). Short-Term Therapy of Experimental Tinea Pedis in Guinea Pigs with Lanoconazole, a New Imidazole Antimycotic Agent. Antimicrob. Agents Chemother..

[146] Niwano Y., Seo A., Kanai K., Hamaguchi H., Uchida K., Yamaguchi H. (1994). Therapeutic efficacy of Lanoconazole, a New Imidazole Antimycotic Agent, for Experimental Cuteneous Candidiasis in Guinea Pigs. Antimicrob. Agents Chemother..

[147] Ogata M., Matsumoto H., Hamada Y., Takehara M., Tawara K. (1983). 1-[1-[2-(3-Chlorobenzyl)oxy]phenyl]vinyl]-1*H*-imidazole Hydrochloride, a New Potent Antifungal Agent. J. Med. Chem..

[148] Ogata M., Matsumoto H., Shimizu S., Kida S., Shiro M., Tawara K. (1987). Synthesis and Antifungal Activity of New 1-Vinylimidazoles. J. Med. Chem..

[149] Torres-Rodriguez J.M., Mendez P., Lopez-Jodra O., Morea Y., Espasa M., Jeminaz T., Lagunas C. (1999). *In Vitro* Susceptibilities of Clinical Yeast Isolates to the New Antifungal Eberconazole Compared with Their Susceptibilities to Clotrimazole and Ketoconazole *Antimicrob*. Agents Chemother..

[150] Enoch D.A., Ludlam H.A., Brown N.M. (2006). Invasive fungal infections: a review of epidemiology and management options. J. Med. Microbiol..

[151] Sahin G.O., Akova M. (2006). Treatment of invasive infections due to rare or emerging yeasts and moulds. Exp. Opin. Pharmacother..

[152] Nucci M. (2003). Emerging moulds: Fusarium, Scedosporium and Zygomycetes in transplant recipients. Curr. Opin. infect. Dis..

[153] Bartoli J., Turmo E., Alguero M., Boncompte E., Vericat M.L., Conte L., Ramis J., Merlos M., Garcia-Rafanell J., Forn J. (1998). New azole antifungals. 2. Synthesis and antifungal activity of heterocyclecarbocamide derivatives of 3-amino-2aryl-1-azolyl-2-butanol. J. Med. Chem..

[154] Bartoli J., Turmo E., Alguero M., Boncompte E., Vericat M.L., Conte L., Ramis J., Merlos M., Garcia-Rafanell J., Forn J.  (1998). New azole antifungals. 3. Synthesis and antifungal activity of 3-substituted-4(3H)-quinazolinones. J. Med. Chem..

[155] Pasqualotto A.C., Thiele K.O., Goldani L.Z. (2010). Novel triazole antifungal drugs: focus on isavuconazole , ravuconazole and albaconazole. Curr. Opin. invest. D.

[156] Fung-Tomc J.C., Huczko E., Minassian B., Bonner D.P. (1998). *In vitro* activity of a new oral triazole, BMS-207147 (ER-30346). Antimicrob. Agents Chemother..

[157] Minassian B., Huczko E., Washo T., Bonner D., Fung-Tomc J. (2003). *In vitro* activity of ravuconazole against zygomycetes, Scedosporium and Fusarium isolates. Clin. Microbiol. Infect..

[158] Warn P.A., Sharp A., Denning D.W. (2006). *In vitro* activity of a new triazole BAL4815, the active component of BAL8557 (the water-soluble prodrug), against Aspergillus spp. Antimicrob. Agents Chemother..

[159] Odds F.C. (2006). Drug evaluation: BAL-8557--a novel broad-spectrum triazole antifungal. Curr. Opin. Invest. Drugs.

[160] Guinea J., Bouza E. (2008). Isavuconazole: a new and promising antifungal triazole for the treatment of invasive fungal infections. Future Microbiol..

[161] Odds F., Ausma J., Van Gerven F., Woestenborghs F., Meerpoel L., Heeres J., Vanden Bossche H., Borgers M. (2004). *In vitro* and *in vivo* activities of the novel azole antifungal agent R126638. Antimicrob. Agents Chemother..

[162] Vanden Bossche H., Ausma J., Bohets H., Vermuyten K., Willemsens G., Marichal P., Meerpoel L., Odds F., Borgers M. (2004). The novel azole R126638 is a selective inhibitor of ergosterol synthesis in Candida albicans, Trichophyton spp. and Microsporum canis. Antimicrob. Agents Chemother..

[163] Meerpoel L., Backx L.J.J., Van der Veken L.J.E., Heeres J., Corens D., De Groot A., Odds F.C., Van Gerven F., Woestenborghs R., Van Breda A., Oris M., Van Dorsselaer P., Willemsens G.H.M., Vermuyten K.J.P., Marichal P.J.M.G., Vanden Bossche H.F., Ausma J., Borgers M. (2005). Synthesis and *in vitro* and *in vivo* Structure-Activity Relationships of Novel Antifungal Triazoles for Dermatology. J. Med. Chem..

[164] Geria A.N., Scheinfeld N.S. (2008). Pramiconazole, a triazole compound for the treatment of fungal infections. IDrugs.

[165] Ryder N.S., Mieth H. (1992). Allylamine antifungal drugs. Curr. Top. Med. Mycol..

[166] Krishnan-Natesan S. (2009). Terbinafine: a pharmacological and clinical review. Expert Opin. Pharmacother..

[167] Denning D.W. (2003). Echinocandin antifungal drugs. Lancet.

[168] Turner M.S., Drew R.H., Perfect J.R. (2006). Emerging echinocandins for treatment of invasive fungal infections. Expert Opin. Emerging Drugs.

[169] Odds F.C. (2001). Sordarin antifungal agents. Expert Opin. Ther. Patents.

[170] Gargallo-Viola D. (1999). Sordarins as antifungal compounds. Curr. Opin. Anti-Infective Invest. Drugs.

[171] Richardson M., Lass-Flörl C. (2008). Changing epidemiology of systemic fungal infections. Clin. Microbiol Infect..

[172] Pfaller M.A., Diekema D.J. (2010). Epidemiology of Invasive Mycoses in North America. Critical Rev. Microbiol..

[173] Bruggemann R.J.M., Alffenaar J.W.C., Blijlevens N.M.A., Billaud E.M., Kosterink J.G., Verwey W.P.E., Burger D.M. (2009). Clinical Relevance of the Pharmacokinetic Interaction of Azole Antifungal drugs with Other Coadministered Agents. Clin. Infect. Dis..

[174] Hardin T.C., Graybill J.R., Fetchick R., Woestenborghs R., Rinaldi M.G., Kuhn J.G. (1988). Pharmacokinetics of itraconazole following oral administration to normal volunteers. Antimicrob. Agents Chemother..

[175] Zhang A.Y., Camp W.L., Elewski B.E. (2007). Advances in Topical and Systemic Antifungals. Dermatol. Clin..

[176] Marichal P., Koymans L., Willemsens S., Bellens D., Verhasselt P., Luyten W., Borgers M., Ramaekers F.C.S., Odds F.C., Vanden Bossche H. (1999). Contribution of mutations in the cytochrome-P450 14α -demethylase (Erg11p, cyp51p) to azole resistance in Candida albicans. Microbiology.

[177] Marichal P. (1999). Mechanisms of resistance to azole antifungal compounds. Curr. Op. Anti-Infect. Invest. Drugs.

[178] Cannon R.D., Lamping E., Holmes A.R., Niimi K., Baret P.V., Keniya M.V., Tanabe K., Niimi M.G.A., Monk B.C. (2009). Efflux-mediated antifungal drug resistance. Clin. Microbiol. Rev..

[179] Peman J., Canton E., Espinel-Ingroff A. (2009). Antifungal drug resistance mechanisms. Exp. Rev. Anti-Infect..

[180] Hof H. (2008). Is there a serious risk of resistance development to azoles among fungi due to the widespread use and long-term application of azole antifungals in medicine?. Drug Resist. Updates.

[181] Ferreira da Silva M.E., Colombo A. L., Paulsen I., Ren Q., Wortman J., Huang J., Goldman M.H.S., Goldman G.H. (2005). The ergosterol biosynthesis pathway, transporter genes, and azole resistance in Aspergillus fumigates. Med. Mycol..

[182] Sanglard D. (2002). Resistance of human fungal pathogens to antifungal drugs. Curr. Opin. Microbiol..

[183] Rex J.H., Pfaller M.A., Galgiani J.N., Bartlett M.S., Espinel-Ingroff A., Ghannoum M.A., Lancaster M., Odds F.C., Rinaldi M.G., Walsh T.J., Barry A.L. (1997). Development of interpretive breakpoints for anti-fungal susceptibility testing: conceptual framework and analysis of *in vitro/in vivo* correlation data for fluconazole, itraconazole, and *Candida* infections. Clin. Infect. Dis..

[184] Odds F.C. (1998). Should resistance to azole antifungals in vitro be interpreted as predicting clinical non-response?. Drug Resist. Updates.

[185] Bates D.W., Yu D.T. (2003). Clinical impact of drug-drug interactions with systemic azole antifungals. Drugs Today.

[186] Girmenia C. (2009). New generation azole antifungals in clinical investigation. Expert Opin. Invest. Drugs.

[187] Wang E.-J., Lew K., Casciano C.N., Clement R.P., Johnson W.W. (2002). Interaction of common azole antifungals with P glycoprotein. Antimicrob. Agents Chemother..

[188] Baciewicz A.M., Baciewicz F.A. (1993). Ketoconazole and fluconazole drug interactions. Arch. Int. Med..

